# Widely-based full-genome analyses enable development of universal and strain-specific PCR toolkit for wheat dwarf virus detection, revealing new alternative hosts and challenging strain-host specificity

**DOI:** 10.1186/s13007-025-01420-6

**Published:** 2025-07-21

**Authors:** Botond Zsombor Pertics, Gergely Tholt, András Kis, Éva Szita, Kornél Gerő, Regina Gerstenbrand, Janka Simon, Ferenc Samu

**Affiliations:** 1 National Laboratory for Health Security, HUN-REN Centre for Agricultural Research Plant Protection Institute, Budapest, Hungary; 2 Department of Zoology, Plant Protection Institute, HUN-REN Centre for Agricultural Research, Budapest, Hungary; 3 Institute of Genetics and Biotechnology, Hungarian University of Agriculture and Life Sciences, Gödöllő, Hungary

**Keywords:** Wheat dwarf virus, *Psammotettix alienus*, WDV host range, WDV strain specificity, WDV wheat strain, WDV barley strain, WDV reservoir, PCR primers

## Abstract

**Background:**

Wheat dwarf virus (WDV) is a destructive cereal virus causing significant yield losses in wheat and barley. It is transmitted by the leafhopper *Psammotettix alienus* and can persist in wild grasses between growing seasons, making reliable detection and strain differentiation critical for disease management.

**Results:**

We developed a comprehensive PCR toolkit for WDV by analysing 38 complete genome sequences, reviewing, validating, and upgrading existing primers and designing new primers spanning multiple viral genome regions. The primer toolkit achieved high diagnostic and analytical specificity as it consistently detected WDV in plants and insect vectors. This enabled the separation of WDV wheat- and barley-strains through a two-step workflow: screening with universal primers, and strain assignment with strain-specific primer pairs. Field testing across 13 Hungarian sites revealed barley strain dominance in the samples, infecting not only barley but also wheat and multiple grass species. Our surveys identified three previously undocumented reservoir grasses adding to the reviewed host range of 42 species. Complete genome sequencing of one wheat-strain and two barley-strain isolates confirmed > 99% intra-strain nucleotide identity but only ~ 85% between strains. Spatial mapping demonstrated virus concentration in grassy islands with declining titers toward cultivated areas, suggesting these serve as infection reservoirs.

**Conclusions:**

This validated primer panel provides a robust framework for studying WDV epidemiology and developing targeted management strategies for this economically important pathogen. Understanding this model of virus-vector system and the improvement of the presented methods are key factors to combat other similarly operating plant-vector-pathogen systems.

**Supplementary Information:**

The online version contains supplementary material available at 10.1186/s13007-025-01420-6.

## Background

### Wheat dwarf virus

**Fig. 1:**
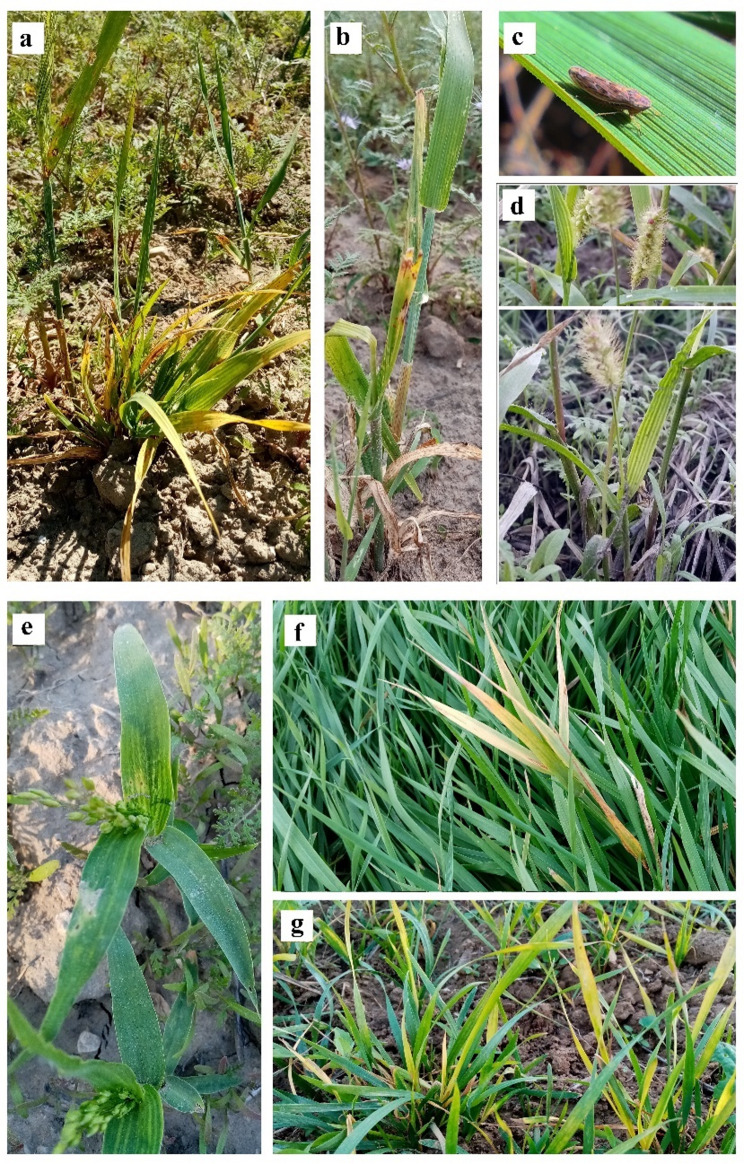
Symptoms of WDV on different plants. **a**-**b**) *Hordeum vulgare*, **d**) *Setaria pumila*, **e**) *Panicum miliaceum*, **f**) *Arrhenatherum elatius*, **g**) *Triticum aestivum.* Visible symptoms are yellowing and/or streaking of the leaves, internode-shortage and heading retention. On barley, all symptoms were observable. On *Setaria* and *Panicum*, only the significant streaking is observable. Pictures **a**, **b**, **d**, **e** were taken at the fallow of site VIII (Vértesboglár) in May (picture **a**: sampling event 19– Fig. S2, **b**: sampling event 20 -Fig. S3) or in September (picture d: sampling event 22 -Fig. S5). Picture **f** and **g** are showing an *A. elatius* and wheat, respectively, with only the yellowing of the leaves, still, both were positive for WDV. Picture **f** is from site XI (Csákvár, sampling event 29– Fig. S8), **g** is from site XIII (Bakonykúti, sampling event 33– Fig. S10). Picture **c**: *Psammotettix alienus* adult, the vector of WDV (at site IIIb Csúza in 2023 November)

Wheat dwarf virus (WDV) (Vacke, 1961) [1], is a widespread and economically significant pathogen of cereal crops, reported across Europe and Asia over the past century [2, 3]. The main host plants of WDV are wheat (*Triticum aestivum*) (Fig. 1g) and barley (*Hordeum vulgare*) (Fig. 1a, b), however, there are indications of infections in related species like rye and oats [4]. The virus causes symptoms such as yellowing (Fig. 1a, f, g) or streaking (Fig. 1a, b, d, e) of leaves, stunted growth, shortened internodes (Fig. 1a, b), and absent head development (Fig. 1a, b), leading to yield losses of up to 80% [2, 3].

The WDV genome is a single-stranded circular DNA (~ 2.7 kb) encoding four proteins: movement protein (MP), coat protein (CP), and two replication-associated proteins (Rep and RepA). It also contains large and small intergenic regions (LIR, SIR), which regulate viral replication and transcription [4]. Conventional phylogenetic studies have classified WDV into two main subgroups: wheat (WDV-W) and barley (WDV-B) strain, based on nucleotide sequence differences and host specificity [5–7]. Further analyses have identified five strains [8]. According to this, strain A infects barley, strain C infects wheat, strains B and D infect barley (both are reported only from Iran), strain E infects wheat and other *Poaceae* including *Lolium* and *Secale* spp. Later, these and a sixth (F) strain based not on host specificity but sequence similarities in the large intergenic region (LIR) were assigned to WDV-barley (strain A and F) or associated with WDV-wheat (strains B, C, D, and E) [4, 9]. A new strain (G) was also suggested later [10]. Other authors argued for an evolutionary split into wheat dwarf virus (WDV), barley dwarf virus (BDV) and oat dwarf virus (ODV) as three different species or subspecies, reflecting coadaptation with their respective hosts [11, 12].

Current classification identifies WDV and ODV as two separate viruses (≈ 70% nucleotide identity) and splits WDV into wheat and barley strains (≈ 80–85% nucleotide identity in between strains, further divided into lesser A-F variants, abbreviated as WDV/A, WDV/B etc.). Strain demarcation threshold at < 94% shared nucleotide identity of full-length nucleotide sequences is supported by the International Committee on Taxonomy of Viruses (ICTV), as well as the use of binomial species names since 2023: *Mastrevirus hordei* and *M. avenae* for WDV and ODV, respectively. Relying upon this and considering that most of the studies follow the traditional division (wheat and barley strains), we will refer them as WDV-wheat and WDV-barley strains, abbreviated as WDV-W and WDV-B, respectively. We refer to the subdivisions WDV/A–F [8, 9] as “substrains”.

In spite of its economic importance, still there are considerable knowledge gaps regarding the infection cycle and epidemics of WDV. The complexity of WDV infections is compounded by its ability to infect multiple host plants creating a dynamic infection cycle that involves both cultivated cereals and natural reservoirs in wild grasses [4, 9, 11]. Mapping this network demands knowledge of which WDV strains circulate in each host and local vector populations, because even though the wheat strain generally infects wheat and the barley strain barley, occasional crossovers—such as barley strains in wheat or vice versa—have been documented [3, 13], but we have little knowledge about the frequency and generality of such cross-infections. Moreover, several wild grasses have emerged as novel hosts for both strains, potentially acting as longterm viral reservoirs [3, 14, 15], but again we are far from knowing the full spectrum of alternative hosts and their significance in WDV epidemics. To fill in the knowledge gaps the detection and differentiation of WDV strains are crucial for managing its spread effectively.

### Virus vector and the epidemiological cycle

The only know vector of WDV is the leafhopper *Psammotettix alienus* (Dahlbom, 1850), a widespread species in cereal fields and grasslands (Fig. 1c). This insect transmits WDV in a persistent, non-propagative manner, meaning the virus remains infectious but does not replicate within the vector. When the vector feeds on an infected plant, the virus is retained within the vector’s salivary glands after crossing the gut epithelium and hemocoel, ensuring its persistence over the insect’s lifespan [2, 16]. Transmission occurs when viruliferous leafhoppers feed on host plants, introducing the virus into the phloem tissue. Both larval and adult stages of *P. alienus* are capable of transmitting the virus, with no significant differences in transmission efficiency based on sex or life stage [2, 16–19]. This efficient transmission process contributes significantly to the widespread distribution and epidemiological impact of WDV in cereal crops such as wheat and barley.

The epidemiological cycle of WDV is tightly synchronized with the life stages of *P. alienus*, and the cultivation patterns of cereal crops. In autumn, adult leafhoppers transmit WDV to newly sown winter wheat (*T. aestivum*) and barley (*H. vulgare*), initiating primary infections [18, 19]. Female leafhoppers lay eggs on cereal leaves, which overwinter in fields and hatch into nymphs the following spring when temperatures exceed 10 °C [20]. These nymphs develop through five larval stages over 32 days before maturing into adults in early summer [20].

Secondary infections occur when nymphs acquire WDV from infected winter cereals and spread the virus locally through feeding, creating patchy infection patterns [18]. In summer, after harvest, adult leafhoppers migrate to wild grasses (e.g. *Apera spica-venti*, *Poa pratensis*) and volunteer cereals, which serve as reservoirs for WDV during non-cropping periods [19, 21]. By autumn, viruliferous adults return to newly planted winter cereals, completing the annual cycle. Wild grasses play a critical role in sustaining WDV and *P. alienus* populations. Temperature thresholds strongly influence vector activity, with optimal transmission occurring above 15 °C, while activity declines below 10 °C [19].

### Molecular detection methods

As WDV is a single-stranded circular DNA virus, molecular detection methods include polymerase chain reaction (PCR), quantitative PCR (qPCR), enzyme-linked immunosorbent assay (ELISA), and loop-mediated isothermal amplification (LAMP). PCR has been widely used for WDV detection due to its high sensitivity and specificity. For instance, duplex PCR with strain-specific primers amplify 734 bp (WDV-W) or 483 bp (WDV-B) fragments in a single reaction [17]. Real-time PCR with TaqMan probes further allows precise quantification in plants and in individual *Psammotettix alienus* vectors [2, 16].

Double-antibody sandwich ELISA (DAS-ELISA) is still favoured for large surveys because it is inexpensive and rapid, although its sensitivity is lower than PCR; extinction values provide a semi-quantitative estimate of virus load [22].

LAMP is emerging as a promising alternative for WDV detection due to its rapidity and simplicity. This method operates under isothermal conditions and does not require sophisticated thermal cyclers, making it suitable for field diagnostics [23]. Pilot assays for WDV have been reported [24–26]. However, its application for WDV detection remains in the experimental phase compared to the more established methods of PCR and ELISA [2].

Regarding PCR primers, earlier studies highlighted challenges in designing effective primers due to issues such as non-specific amplification and low product yield. For example, initial primer pairs like SeqvF/SeqvR failed to produce the expected 2.7 kb amplicon of the full WDV genome, leading to extensive redesign efforts. Subsequent combinations such as Bar12/SeqvR2 and Bar12/Bar13 showed improved performance but still required optimization to reduce background noise and smearing during gel electrophoresis [27]. More recently, rolling circle amplification (RCA) has been combined with PCR to enrich viral DNA prior to amplification, improving the reliability of detection protocols [22]. However, designing general primers that detect every WDV strain, as well as creating reliable strain specific primers remain tasks to be solved.

### Objectives of the present study

The work presented here had three aims.

1. Assembling a new PCR primer toolkit. Given, that many existing primers were found unreliable, we aimed to overview and evaluate existing primers and design new robust generic and strain-specific sets for routine diagnosis;
2. Surveying WDV spatio-temporal dynamics and host plants. We intended to test the new primer toolkit in a pilot survey of WDV prevalence and phenology in Hungary, paying special attention to alternative host plants, such as wild grasses and volunteer crops;
3. Exploring WDV genetic diversity. As part of proving the robust applicability of the new primers, we aimed to sequence representative Hungarian WDV isolates and confirm their detectability and phylogenetic placement.

## Materials and methods

### Sampling sites and collection methods

**Fig. 2:**
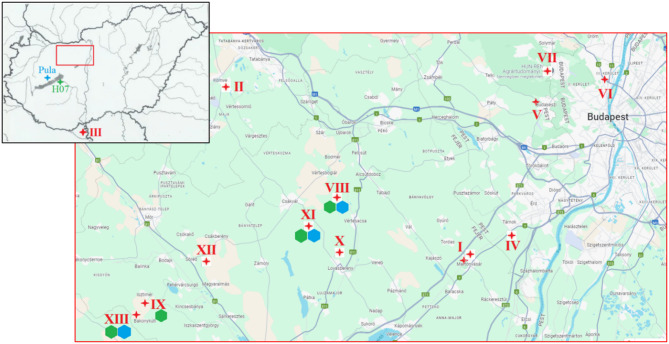
Collection sites of WDV samples in Hungary. The red rectangle on the upper map enframes the more frequent collection sites of the middle-transdanubian region, detailed below. Roman numbers correspond to site numberings in Table S1. GPS coordinates of the sites are listed in Table S3. Hexagons are indicating WDV presence on the site, as a result of this study. Blue: WDV-W, green: WDV-B. Original collection site of WDV reference strains ‘Pula’ and ‘H07’ are also indicated (Pula and Siófok, respectively)

Between April 2023 and October 2024 we visited 13 locations in the Middle-Transdanubian region of Hungary (Fig. 2; Table S1). Thirty-three sampling events (Table S2) captured all critical phases of the cereal-growing calendar; supplementary site and sampling descriptions are provided in Note S1.

Leafhoppers were collected with a modified Husqvarna 125 BV E-tech blower-vac that operated as a lightweight suction sampler [28]. Captured insects were tipped into finemesh bags and either (i) frozen at − 20 °C–− 40 °C for subsequent molecular work or (ii) transported alive for colony establishment and transmission tests. In the laboratory all specimens—live or frozen—were examined under a stereomicroscope; only *Psammotettix alienus* individuals were retained, because this species was the sole focus of the study.

At each collection site plant material was taken in parallel. Two approaches were used: (1) leaf fragments (≈ 1 × 1 cm) clipped with sterile scissors, sealed in sterile bags and frozen at − 20 °C; (2) whole plants (barley, wheat or wild grasses) carefully uprooted, potted and carried to the glasshouse for detailed morphological examination and testing for WDV. The full chronology of sampling events and local conditions is summarised in Table S2 and expanded in Note S1.

### Management of leafhopper stock populations

Rearing of *Psammotettix alienus* largely followed the protocol of [29]. Barley (*Hordeum vulgare* cv. MV Conchita) was the routine host plant. Seeds were sown in ‘Jóföld– Palántaföld’ potting compost (Pax96 Ltd., Kecskemét, Hungary) and grown in a greenhouse to the advanced tillering stage. Pots were then moved to a controlled-environment chamber set to 23 °C (day) / 16 °C (night) with a 16 h light / 8 h dark photoperiod. Each pot was enclosed in a fine nylon-mesh cage that confined the leafhoppers while allowing ample airflow. Insects were transferred to fresh barley every fortnight, or sooner if the plants became exhausted. To maintain genetic diversity, the colony was supplemented once a year with adults collected from several field sites.

For selected experiments, wheat (*Triticum aestivum*) replaced barley. Seed came from Martonvásár (Rozsdakert, Site Ia) and was raised under the same environmental conditions. Additional trials employed wild grasses—downy brome (*Bromus tectorum*) and soft brome (*B. hordeaceus*)—collected at Vértesboglár (Site VIII, Event 20), which were established in pots and used as alternative host plants.

#### Establishment of viruliferous vector colonies

Field collections from Martonvásár (event 17), Vértesboglár (21), Isztimér (24), Csákvár (29) and Söréd (31) were introduced into mesh-cage rearings in the glasshouse (Fig. S14). Host barley plants were screened fortnightly with PCR. 

In the laboratory only two stable virus lines could persist: the long-standing laboratory strain “vteny” (WDV-B) and a WDV-B colony founded with vectors from Csákvár. All attempts to establish WDV-W colonies failed. Lines begun with vectors from Vértesboglár and Isztimér occasionally produced WDV-B positive plants but quickly lost the virus, as did the mixed “MV” line established with Martonvásár and Környe insects; in that case the infection source could not be traced.

### DNA extraction

Frozen insects (individual or pooled) and ~ 1 × 1 cm leaf fragments were placed in 1.5 ml microcentrifuge tubes. Each tube received 100–200 µl Extraction Solution (Sigma-Aldrich E7526) and the tissue was homogenised with a disposable plastic pestle (Bel-Art Products/SP, USA) until fully dispersed. Samples were heated at 95 °C for 10 min to complete lysis, cooled briefly, then mixed with 100 µl ExtractNAmp Plant Dilution Solution (Sigma-Aldrich D5688) and vortexed. One microlitre of the clarified lysate served directly as template for PCR or qPCR.

### Reference genomes and primer design

**Primer-design workflow.** We assembled 38 complete WDV genomes from the literature [9, 24, 25, 30–31]: 17 barley strain sequences (substrains WDV/A, B, F and unclassified variants) and 21 wheat strain sequences (substrains WDV/C–E and unclassified variants) (Table 1). Accession numbers were taken from the original papers. All genomes were aligned with BLASTn (multiFASTA output) and examined in Jalview 2.11.4.0.

Published primer sets [3, 6, 30, 32] were synthesised (SigmaAldrich) and screened first (Table 2). New primers were then designed manually on the multiple-sequence alignment:

**Table 1:** List of WDV sequences used in this study. Sequences available in the database were used for multiple sequence alignment to design primers. Grey background indicates the three WDV samples sequenced during this study. Substrains ‘A-F’ of strains (wheat/barley) are indicated where they are known. References, from which the accession numbers were obtained are indicated, T: This study

	accession number	WDV strain, substrain	host plant, origin	ref.
1	FM210034.1 (H07)	barley	*H. vulgare*, Hungary	[33]
2	FN806786.1 (Pula)	wheat	*T. aestivum*, Hungary	[34]
3	AJ311031	wheat	*T. aestivum*, Sweden	[3, 31]
4	AJ311035	wheat E	*T. aestivum*, Sweden	[31]
5	AJ311037	wheat E	*T. aestivum*, Sweden	[31]
6	AJ783960	barley A	*H. vulgare*, Turkey	[24]
7	AM040732	wheat E	*H. vulgare*, Hungary	[24]
8	AM296022	wheat E	*Lolium sp.*, Germany	[31]
9	AM296024	barley F	*T. aestivum*, Germany	[25]
10	AM411651	barley F	*H. vulgare*, Germany	[25]
11	AM491478	wheat E	*A. fatua*, Sweden	[31]
12	AM491481	wheat E	*A. spica-venti*, Sweden	[31]
13	AM491482	wheat E	*P. pratensis*, Sweden	[31]
14	AM491486	wheat E	*L. multiflorum*, Sweden	[31]
15	AM491488*	wheat E	*P. alienus*, Sweden	[31]
16	AM747816	barley	*H. vulgare*, Hungary	[25]
17	AM980882	barley A	*H. vulgare*, Germany	[31]
18	AM989927	barley A	*H. vulgare*, Bulgaria	[25]
19	EF536880	wheat	no data, China	[9, 25]
20	EF536881	wheat	no data, China	[9, 25]
21	FJ546179	barley A	*H. vulgare*, Czech Rep.	[25]
22	FJ546181	barley F	*H. vulgare*, Germany	[25]
23	FJ620684	barley B	*H. vulgare*, Iran	[24, 31]
24	FM999833	barley A	*H. vulgare*, Hungary	[31]
25	FN806784	wheat E	*T. aestivum*, Ukraine	[31]
26	FN806785	wheat	*H. vulgare*, Hungary	[31]
27	FN806787	barley A	*T. aestivum*, Ukraine	[31]
28	HF968639	barley A	*H. vulgare*, Spain	[25]
29	HF968641	barley A	*H. vulgare*, Spain	[25]
30	HF968646	barley A	*H. vulgare*, Austria	[25]
31	JN791096	wheat D	*H. vulgare*, Iran	[24, 31]
32	JQ647455	wheat C	*T. aestivum*, Hungary	[24, 31]
33	JQ647508	wheat C	*T. aestivum*, China	[31]
34	KM079154	wheat	*H. vulgare*, Poland	[24]
35	KM079155	barley	*H. vulgare*, Poland	[24]
36	MN453813	wheat E	*Lolium sp.* Sweden	[31]
37	MN594280	barley A	*H. vulgare*, France	[3]
38	MN594281	wheat E	*T. aestivum*, France	[3]
	PV683564 (vteny)	barley	*P. alienus*, Hungary	T
	PV683562 (vbogK1)	barley	*P. alienus*, Hungary	T
	PV683563 (vbogK14)	wheat	*P. alienus*, Hungary	T

*Accession number for this sequence was erroneously given in the paper as ‘AM491498’ directing toa different organism (*Botrytis cinerea*)

**Table 2:** Primers used in this study for WDV identification. Original primers and their modified counterparts are in the same line. Bold letters in the sequences indicatenucleotides changed compared to the original primers. Primer orientation in the WDV full genome and primer codenumbers are indicated on Fig. 3. All primers were applied using PCR cycle A*. Performance: W-working in vitro, NW-not working in vitro, NSS-not strain-specific in vitro, NP – not perfect on nucleotide level (in silico), R – recommended to use. References are indicated, T-this study

	target		ORIGINAL				MODIFIED			
ref.	strain	region	code	primer name	5’-3’sequence	performance	code	primer name	5’-3’sequence	performance
(30)	WDV-W			WDV-2730	CCRCACACCDAACASGGCCCA	NW				
(30)	WDV-W			WDV-1430	GAAYGAGTAGTTGATGAAYGWCTC	NW				
(30)	WDV-B			WDV-2720	CGCGGGACCACCCGTCGCT	NW				
(30)	WDV-B			WDV-1370	GCGAARAAYGATTCMCCYTCATA	NW				
(32)	WDV	RepA	R	WDVrepDetR	CGCCTTGGACTCTCTTCGCAC	W, NP	91	WDVrepDetDeg-Rev	CGCCTTGGA**B**TC**W**CTTCGCAC	W, R
(32)	WDV	RepA	F	WDVrepDetF	GACGGATAGACCATTCAAACG	W, NP	92	WDVrepDetDeg-For	GACG**K**AT**W**GACCA**K**TCAAACG	W, R
(3)	WDV	CP	7	WFb	CCACTGACATCTTTACGATGC	W, NP	93	WDV-WFb-Deg	CCACTGACAT**M**TTTACGATGC	W, R
(3)	WDV	Rep	8	WRb	GGAAAGACTTCCTGGGCAAG	NW				
(3)	WDV-W	Rep	9	1F	GAGGCGAACGAGTAGTTGAT	W, R				
(3)	WDV-B	Rep	10	4R	AGGGTGAATCATTCTTCG	W, R				
(6)	WDV	MP	30	P1	GACCGAGGAAATTGGTTACGG	W, R				
(6)	WDV-B	CP	31	P3	CACATACAACTTCGAAGTAAA	W, NP, R	89	cp1360-WDV-B_R	**GG**CACATACAACTTC**R**AA**TGM**AA**TGGAGG**	W, NSS
(6)	WDV-W	CP	32	P4	CACATACAACATCAAACGCCG	W, NP	90	cp1360-WDV-W_R	**TAGG**CACATACAACATCAAACGCCG**TAGA**	W, R
			***NEW***							
T	WDV-B	Rep	87	Rep1914-WDV-B_F	TCRRCTAGGCYGTTGTAGTAGTTGTGGA	W, R				
T	WDV-W	Rep	88	Rep1914-WDV-W_F	TCAACTAGACTGTTATAATAATTRTGTG	W, R				
T	WDV	CP	94	cp1260-WDV_R	TATCTTGCCGTCACCCGTGTTCAT	W, R				
T	WDV-W	Rep	95	NFB17 H4	CATCTTCGCCGGAGGCGAACGAG	W, R				
T	WDV	RepA	96	NFB17 H5	ACGATTATTCCAGGAGTCACCGGGG	W, R				

**Fig. 3:**
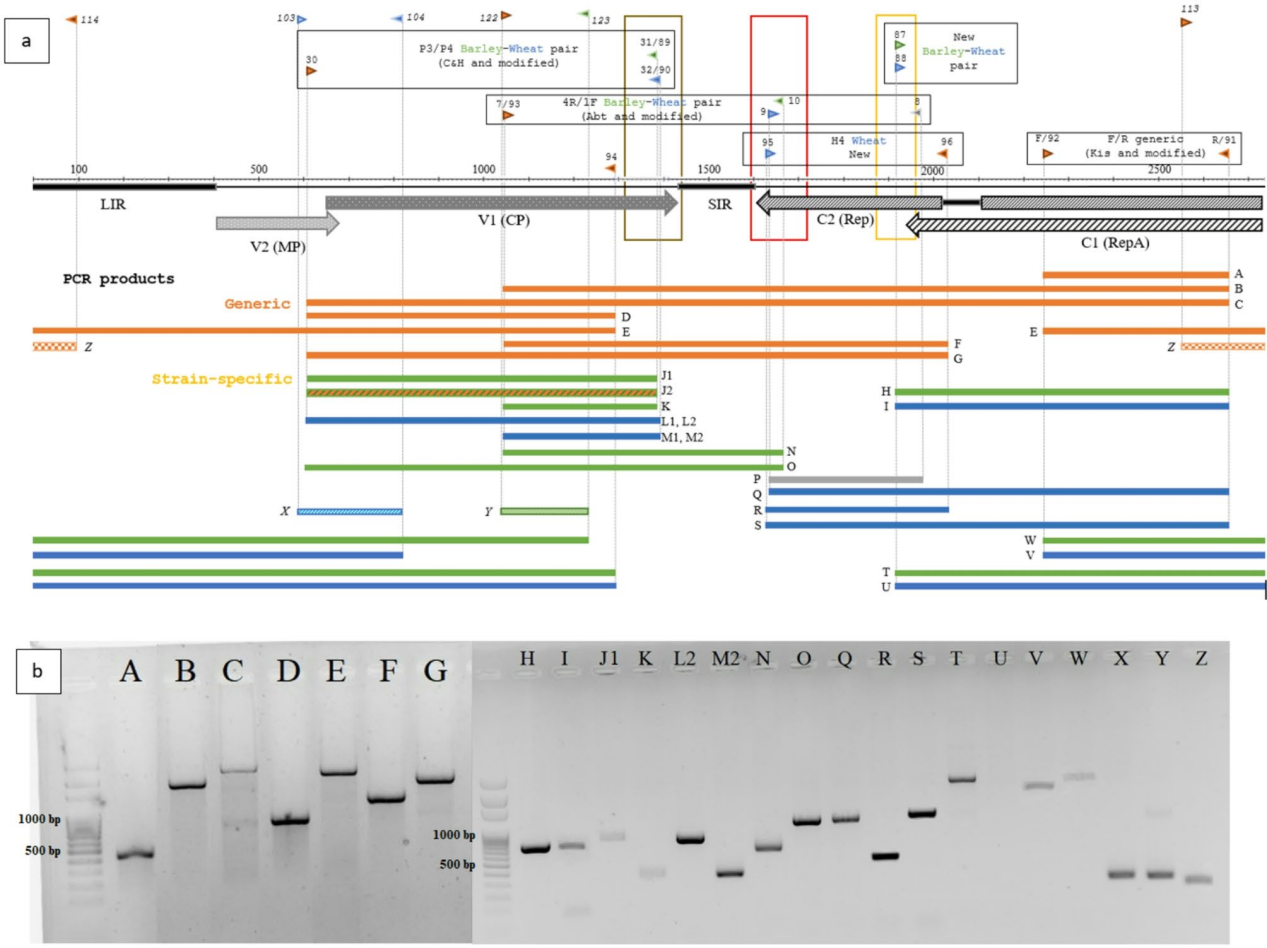
Primers and PCR amplicons for WDV. (**a**) WDV genome organization and the location of the PCR primers. Code numbers of the primers are indicated and expanded in Table 2. Blue triangles stand for wheat-strain specific, green triangles for barley-strain specific, orange triangles for universal primers. Universal PCR amplicons (orange bands) and strain-specific amplicons are marked (wheat: blue bands; barley: green bands). The genome segments surrounded by the brown, red and yellow rectangle are detailed in Fig. 4. Italic primer numbers and amplicons X, Y, Z are indicating outer primers (F3, B3) used in a LAMP reaction in our different study. (**b**) Results of agarose gel electrophoresis of PCR amplicons in alphabetical order. Letters correspond to the amplicons in **a**). A-G: amplicons of generic primers on the template “vteny” (WDV-B strain). H-Z: Amplicons of strain-specific primers. Barley or wheat strain-specific amplicons were amplified either on K1 (WDV-B) or K14 (WDV-W). Amplicon “Z” is generic. M = molecular weight marker

Original primers and their modified counterparts are in the same line. Bold letters in the sequences indicate nucleotides changed compared to the original primers. Primer orientation in the WDV full genome and primer code numbers are indicated on Fig. 3. All primers were applied using PCR cycle A*. Performance: W-working in vitro, NW-not working in vitro, NSS-not strain-specific in vitro, NP– not perfect on nucleotide level (in silico), R– recommended to use. References are indicated, T-this study.*PCR CYCLE A: 15 min 95 °C, 32 cycles of 30 s 95 °C, 30 s 50 °C, 1:30 min 72 °C, followed by 10 min 72 °C

- sites conserved in every isolate → **generic** primers;
- sites conserved only in wheat strain isolates → **WDV-W-specific** primers;
- sites conserved only in barley strain isolates → **WDV-B-specific** primers.

Where possible, primers were placed so that each strain-specific set captured all intra-strain variation yet retained mismatches to the opposite strain. Degenerate positions were introduced sparingly and every candidate was checked in OligoEvaluator (Sigma-Aldrich, www.oligoevaluator.com) for melting temperature, self-dimer and hairpin formation. Finally, we ensured that the full panel covered widely separated regions of the genome (CP, MP and Rep) to minimise false negatives caused by local mutations. 

### PCR, qPCR and sequencing procedures

#### Conventional PCR

All reactions were run on an Arktik Thermo Cycler (Thermo Scientific) in 25 µl volumes containing 5 µl 5X HOT FIREPol Blend Master Mix (Solis BioDyne), 0.25 µl of each primer (10 µM), 18.5 µl H₂O and 1 µl template. Cycling parameters for the three standard programmes (A–C) are listed in Table 2 and S4. All PCR reactions were performed in triplicates and the results were evaluated based on the mean of the three outcomes.

*Generic screen.* Unknown samples were first tested with the generic Rep pair WDVrepDetDeg-For (92) / -Rev (91) (Cycle A, amplicon A, Fig. 3).

**Fig. 4:**
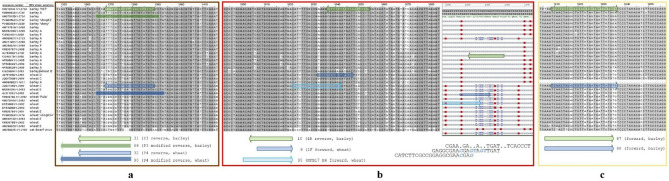
The three hotspots in the WDV genome containing strain-specific primer sites. Multiple sequence alignments of the whole-genome by Jalview. WDV barley and wheat strains are arranged above and below, respectively, strains and substrains are indicated next to the accession numbers. Sequences isolated in this study and an ODV as an outgroup reference are included. Grey background indicates conserved nucleotides between the isolates. Positions in the whole genome are marked on Fig. 3 with a brown (**a**), red (**b**) or yellow (**c**) rectangle. Primer sites are represented by arrows (barley-specific: green, wheat-specific: blue). **a**: Site of the 3’ end of the CP gene. Original P3-P4, and longer, modified, more generic primers are compared. **b**: Site of the 5’ end of the rep gene. Right: multiple sequence alignment of the same site by NCBI MSA viewer, nucleotide differences are indicated by red background. A frequent nucleotide insertion is observed only in the wheat strains, represented by blue clamps (and blue letters below the picture). **c**: Site of the new primers in the rep gene

*Strain assignment.* Positive lysates were then examined with three independent assays (Cycle A, Table 2; Figs. 3 and 4):

1. Rep1914-WDV-B_F (87) + 91 for barley or Rep1914-WDV-W_F (88) + 91 for wheat (amplicons H, I);
2. P1 (30) + P3 (31) for barley or P1 + cp1360-WDV-W_R (90) for wheat (amplicons J1, L2);
3. WDV-WFb-Deg (93) + 4R (10) for barley, or 1F (9) + 91 for wheat (amplicons N, Q).

Amplicons were resolved on 1% agarose gels (5–8 µl load). Plasmids (pZP201) carrying complete genomes of isolates Pula (wheat, FN806786.1) and H07 (barley, FM210034.1) served as reference controls in vitro, together with the in-house barley strain “vteny”. To assess analytical (cross-reactivity) and diagnostic specificity, these were used as materials with known infection status: all three as true positives for generic WDV test, Pula as true positive/negative for wheat/barley strain specific primers, H07 as true positive/negative for barley/wheat strain specific primers, respectively. To further assess analytical (interference) and diagnostic specificity, non-WDV DNA samples (virus-free leafhopper, plants) were used in vitro, and an ODV isolate sequence (AM296025) served as an outgroup/negative control for primer testing in silico. All primers were checked for non-specific hits in the NCBI BLAST database.

#### Species confirmation

In addition to the conventional morphological methods, leafhoppers were verified with primers Psam268F/Psam435R; wheat and barley with MBK primers (Cycle B, Table S4). Indistinct grasses (indefinable by traditional morphological analysis and by using Flora Incognita smartphone application (https://floraincognita.com/) were barcoded with universal markers: ITS (ITS1-F/ITS1-R), ETS (RETS-B1F/RETS-B2F + 18SR) and matK (matK-xf_FwmatK-MALP_Rev) using cycle C. Products were purified (NucleoSpin Gel & PCR Clean-up, Macherey-Nagel) and Sanger-sequenced (Macrogen); identities were confirmed with NCBI BLASTn.

#### Quantitative PCR

For quantitative detection, total DNA extracted from plant materials (from Site XIII, Event 33) was subjected to qPCR on a LightCycler 96 instrument (Roche, Switzerland). For standard curve determination, reference H07 was diluted 10-fold and measured in triplicates. Reactions included 4 µl HOT FIREPol EvaGreen qPCR Mix Plus (5x) (Solis Biodyne), forward and reverse primers 92 − 91 (0.25 µL each), 14.5 µl dH_2_O and 1 µl template in a final volume of 20 µl. The cycling protocol involved 95 °C for 15 min, followed by 50 cycles of 95 °C for 30 s, 50 °C for 30 s, and 72 °C for 1:30 min, concluding with a final extension of 72 °C for 5 min. For detection, the 470/517 nm channel (SYBR Green I) was employed. Data was subsequently analyzed using the instrument’s dedicated software (LightCycler 96, version 1.1). Threshold level was set according to the Cq values of the standards.

#### Whole-genome sequencing and assembly

Three WDV isolates were sequenced: vbogK1 (barley strain), vbogK14 (wheat strain) from leafhoppers collected at site VIII (Vértesboglár) and “vteny” (barley strain), isolated from our in-house rearing. Overlapping fragments (Fig. 3a) were amplified: B, C, E–H, J1, O, T for K1; B, C, E, F, G, I, L2, M2, Q, V, X, Z for K14; A–G, N, K, Y, Z for “vteny”, purified with the QIAquick Gel Extraction Kit (Qiagen) and Sanger-sequenced by Macrogen. Forward and reverse reads were trimmed in chromatogram view, merged, and aligned against reference genomes H07 (barley) or Pula (wheat) in BLASTn; output was saved as “flat queryanchored with dots for identities” (line length 150).

Consensus sequences were built by copying the reference and manually inserting supported nucleotide differences. Discordant positions were revisited in chromatograms until resolved. Genome annotation was performed by GeneMarkS (https://exon.gatech.edu/GeneMark/genemarks.cgi, accessed on 23rd May, 2025). Final genomes were compared with public records in BLASTn and VIRIDIC (https://rhea.icbm.uni-oldenburg.de/viridic/) for pairwise identity and incorporated into a phylogeny with 38 published WDV sequences using the MEGA12 software. This pipeline provided robust placement of the new Hungarian isolates within the barley and wheat strains.

## Results

### Designing new primers

We screened published primers (Table 2) against all 38 reported full genome sequences (Table 1) available at the time of writing of this manuscript.

*Non-working primers.* We excluded after testing the degenerate pairs WDV-2730/1430 and WDV-2720/1370 [30]—which target Rep-region variants—and the generic reverse primer WRb (8), as none produced amplicons with any forward primer in our library (Fig. 3). These primers were ordered and tested in vitro with various WDV samples, but remained dysfunctional, nonetheless.

*Generic primer*s. A few nucleotide changes were performed in some of the original generic primers taking all 38 WDV sequences into account. Eventually, six generic primers—P1 (30), WDV-WFb-Deg (93), cp1260-WDV_R (94), NFB17 H5 (96) and the degenerate pair WDVrepDetDeg-For (92)/-Rev (91)—functioned consistently in every strain-specific or generic combination tested (Fig. 3).

*Modified strain-specific primers *(Fig. 4a)*.* Improved reverse primer P3, named cp1360-WDV-B_R (89) generated amplicons (Fig. 3, J2) on WDV-W strains as well, so it was not effective as a selective primer. The reason for this is unclear, since it targets a triplet (bases 1368-70) that is consistently ‘TTT’ for barley and ‘CGG’ for wheat strains (Fig. 4a), the primers also include three other specific sites. For this reason, we abandoned the use of this primer and returned to the use of the original P3, which was, however, impaired in silico on the nucleotide level, but worked in vitro (Fig. 3, J1). Improved P4, named cp1360-WDV-W_R (90) worked in vitro as well (Fig. 3, L2, M2).

*Unmodified strain-specific primers* (Fig. 4b). Some previously published strain-specific primers required no modification: the 1F/4R (9/10) pair of Abt et al. exploits the wheat strain specific insertions near the Rep 5′ end, and the new NFB17 H4 (95) primer targets two adjacent SNPs near this insertion hotspot.

*New strain-specific primers* (Fig. 4c)*.* To improve coverage, we created two new forward primers on the Rep gene, Rep1914-WDV-B_F (87) and Rep1914-WDV-W_F (88), each bearing seven strain-specific bases; both performed reliably (Fig. 3, H, I). Wheat strain-specific primer NFB17 H4 (95) along with the generic NFB17 H5 (96) are also reported first in this study.

***Recommended primers.*** As a result of our analysis, we suggest the use of the following primers (marked with an ‘R’ in Table 2) in combination with each other, depending on the preferred genome segment (Fig. 3). Generic, forward: P1, WDV-WFb-Deg, WDVrepDetDeg-For, generic reverse: NFB17 H5, WDVrepDetDeg-Rev, cp1260-WDV_R. Strain-specific, forward: Rep1914-WDV-B_F (barley), Rep1914-WDV-W_F (wheat), 1F (wheat), NFB17 H4 (wheat). Strain-specific, reverse: P3 (barley), cp1360-WDV-W_R (wheat), 4R (barley).

Our PCR assay has high analytical specificity, as all reactions were positive to the reference Pula and H07 strains with WDV-W and WDV-B specific primer pairs, respectively, while no cross-reactivity was observed (except for the above-mentioned primer 89) when a strain-specific primer pair was tested on the different strain (field samples and reference samples). Our assay has high diagnostic specificity, as false positives are unlikely to appear: non-WDV negative controls (virus-free plant or leafhopper sample or no template controls) showed no positive results for any of the WDV primers (no interference). All primers were checked in silico and only WRb (8) was specific to the sequence of an oat dwarf virus isolate (AM296025). No non-specific BLAST hits were obtained in the database for any of the primers. The assay also has high diagnostic sensitivity, as it uniformly gave positive results to samples that once were positive, when the tests were repeated. This is acutely true for the strain-specific primers, as they never had false negatives once a sample was declared positive.

### WDV spatio-temporal dynamics and host plants at three hot-spot sampling sites

**Fig. 5:**
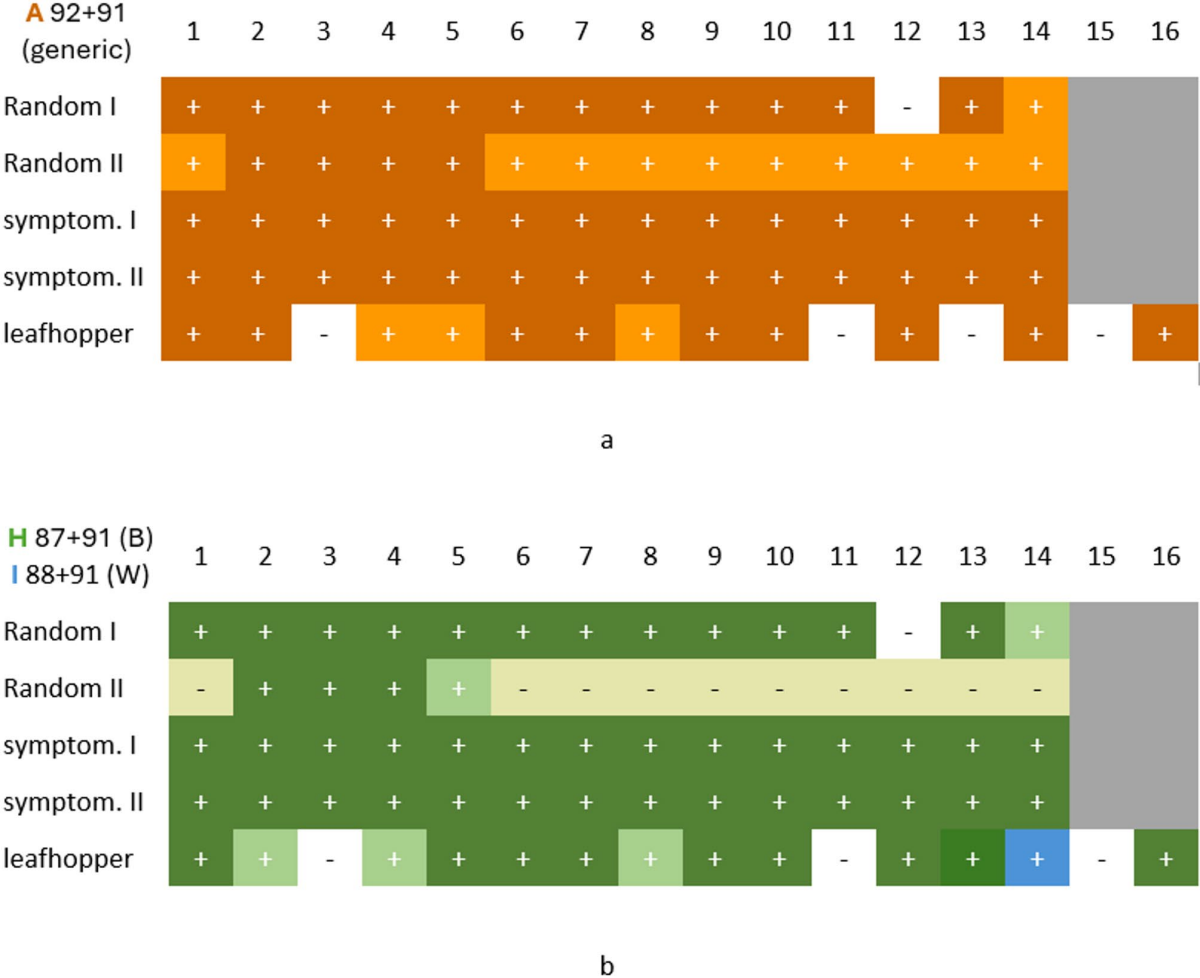
Results of the transect sampling at site VIII (Vértesboglár). Two sets of randomly selected barley samples (14 each), two sets of symptomatic barley samples (14 each) and 16 individual leafhopper samples (K1-K16) were tested for WDV (9 May, event 19, Fig. S2). (**a**) PCR results with generic primers. Dark orange (+) represents strong bands on the agarose gel, light orange (+) means weaker bands. (**b**) The same samples tested with barley strain-specific primers (green). Yellow boxes indicate WDV + samples negative both for WDV-B and for a subsequent WDV-W test, blue box indicates a sample (leafhopper ‘K14’), positive for wheat-specific primers only. Further testing results are shown in Table S5

Observations at Site VIII (Vértesboglár), where transect samples of leafhoppers and plant material were taken (Fig. 5) showed a spring epidemic on self-sown barley peaking on 9 May (27/28 randomly chosen plants were WDV-B positive). There was a sporadic spillover into alternate grasses and a viruliferous leafhopper population in the late May and late July samples. This evolved into a widespread WDV-B infection in wild grasses by late September and early October in the peripheral vegetation after vector disappearance. Wild grasses were tested positive for WDV: *Cynodon dactylon*,* Panicum miliaceum*,* Setaria pumila*,* Lolium* sp. Detailed account of infection dynamics in different hosts is in Note S2, where Supplementary figures are cited referring plant phenology and habitat conditions.

**Fig. 6:**
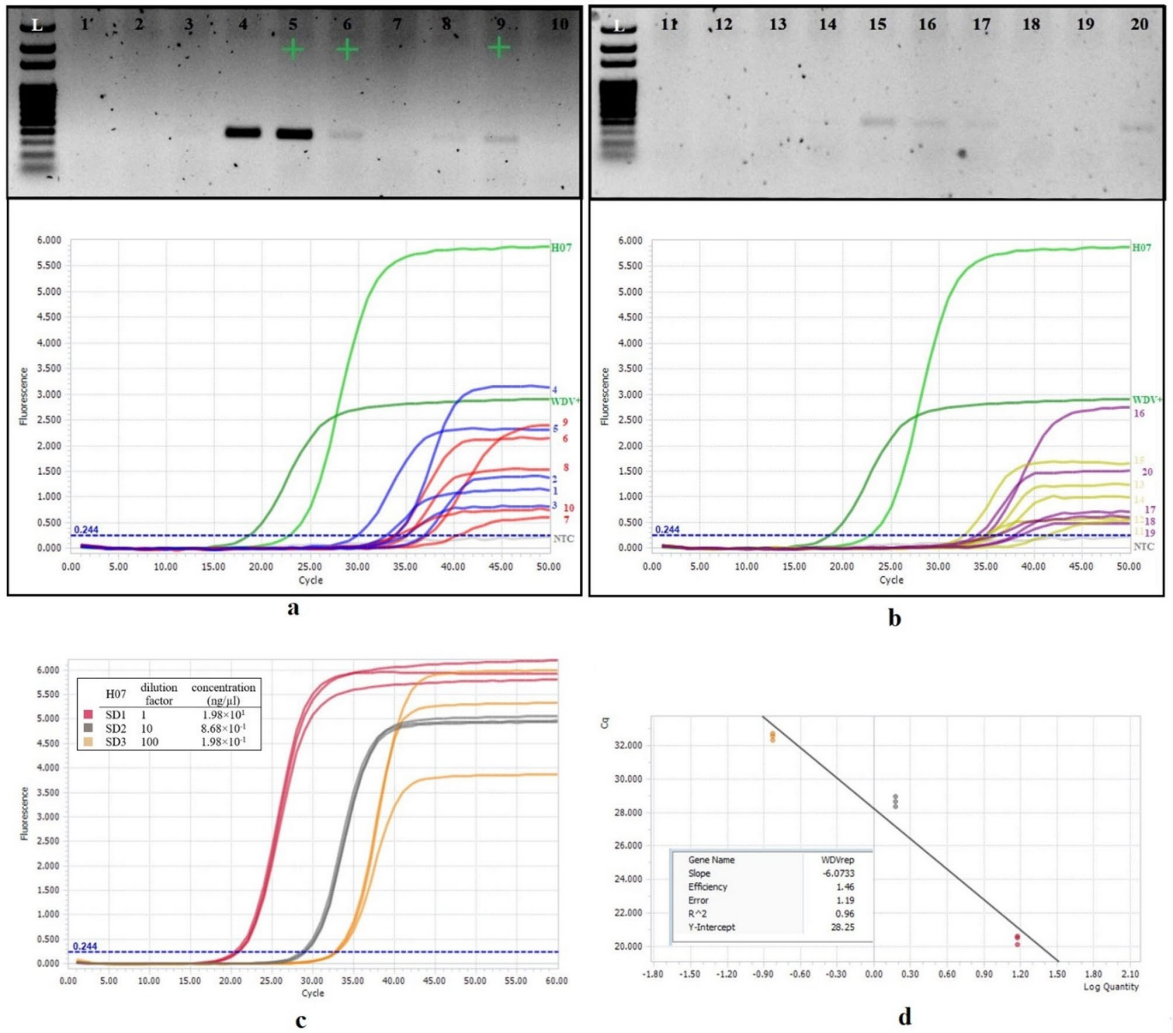
Results of the PCR and qPCR of the ‘A’ amplicon for the samples of the linear gradient at site XIII (Bakonykúti). Samples from 1 to 20 were gathered on a diagonal line across the wheat field, 10 steps in between the sampling points (Fig. S10). **a**-**b**) Up: agarose gel captures of the PCR results. Green crosses indicate positive results for a subsequent WDV-B test (see Table S6 for details). L– molecular ladder. Down: relative qPCR results. 5–5 sample lines were colored the same for better transparency. H07 and a leafhopper sample from stock ‘vteny’ (‘WDV+’) are positive controls. **c**) Amplification curves for the dilution series of sample H07 used as standards for calibration. Initial concentrations are denoted. **d**) Standard curve, log quality is plotted against Cq values for the standards

The sites XI (Csákvár) and XIII (Bakonykúti) were sampled twice in October, about 2½ weeks apart. Although site XI was an abandoned field that was later ploughed, and site XIII an actively managed winter wheat crop, both followed the same trajectory. In early October the virus was confined to vectors. At both sites pooled leafhopper sample contained both strains, whereas all wheat and grass samples from the field interior, grassy margin and a neighbouring fallow were virus-negative. Two to three weeks later, infection had moved into plants while vector prevalence declined. Both strains appeared at least once at each site. On 30th October a transect sample was taken at site XIII., followed by PCR and qPCR mapping (Fig. 6). Virus titres showed a spatially correlated infection patches, with curve along the transect, with highest relative virus titres around sampling points 4–5, secondary foci at 9 and 15–16, and negligible titres at 1–2, 10–13 and 18–19. At these sites of three margin grasses, *Arrhenatherum elatius* harboured a mixed infection, whereas a different *A. elatius* sample and a *Bromus* sp. were negative. *P. alienus* and plant species identity were PCR-confirmed (Fig. S9). Detailed account of infection dynamics in different hosts is in Note S2.

To summarize available information about WDV host plants, in Table 3 we lists all plant species that have been reported as hosts of WDV infection at least once. Present study found WDV in five wild grass species and provided the first evidence of WDV presence in yellow foxtail (*Setaria pumila*) and false oat-grass (*Arrhenatherum elatius*).

**Table 3:** List of hitherto published plants as WDV reservoirs, excluding *T. aestivum* and *H. vulgare*

ref.	plant name	WDV	ref.	plant name	WDV
^1 (30)^	*Aegilops kotschyi*	*+*	^(43), T^	*Panicum miliaceum*	+
^3 (33)^	*Agrostis stolinifera*	*+*	^(37)^	*Phalaris arundinacea*†	+
^(31) (4),5 (38)^	*Apera spica-venti*	*+*	^(15)^	*Phalaris brachystachys*	+
^T^	***Arrhenatherum elatius***†	+	^(33)^	*Phleum pratense*†	+
^9 (15)^	*Avena barbata*	*+*	^(31), (4), (37)^	*Poa annua*	+
^(30), (31), (4) (38),8 (37)^	*Avena fatua*	*+*	^(31), (4), (38)^	*Poa pratensis*†	+
^(31),(33), (4), (15)^	*Avena sativa*	*+*	^(33)^	*Puccinella distans*	+
^(4), (37)^	*Avena sterilis*	*+*	^(4)^	*Secale cereale*	+
^(33), (4)^	*Avena strigosa*	*+*	^T^	***Setaria pumila*** *	+
^3 (33), (37)^	*Bromus arvensis*	*+*	^3 (33)^	*Setaria italica*	+
^(30), (37)^	*Bromus commutatus*	*+*	^(30), (15)^	*Sorghum halepense*	+
^8 (37)^	*Bromus hordeaceus*	*+*	^(22)^	*Triticum spelta*	+
^(4), (37)^	*Bromus inermis*†	*+*	^(4)^	triticale	+
^(37)^	*Bromus japonicus*	*+*	^(37)^	*Zea mays*	+
^(15)^	*Bromus rubens*	*+*	^(30)^	*Agropyron repens*	**-**
^(4), 8 (37)^	*Bromus secalinus*	*+*	^(38)^	*Agrostis canina*	**-**
^(37)^	*Bromus sterilis*	*+*	^(33), (38)^	*Alopeculus pratensis*	**-**
^(4), (37)^	*Bromus tectorum*	*+*	^(33), (38)^	*Arrhenatherum elatius*†	**-**
^(30), T^	*Cynodon dactylon*	*+*	^(38)^	*Brachypodium pinnatum*	**-**
^(33)^	*Dactylis glomerata*†	*+*	^(33)^	*Bromus erectus*	**-**
^(30)^	*Eremopoa persica*	*+*	^(33)^	*Bromus inermis*†	**-**
^(15)^	*Hordeum glaucum*	*+*	^(38)^	*Dactylis glomerata*†	**-**
^(30), (4)^	*Hordeum murinum*	*+*	^(38)^	*Deschampsia cespitosa*	**-**
^(30)^	*Hordeum spontaneum*	*+*	^(38)^	*Elymus repens*	**-**
^(4), (37)^	*Lagurus ovatus*	*+*	^(33), (38)^	*Festuca pratensis*	**-**
^(31) (33),4 (4)^	*Lolium multiflorum*†	*+*	^(38)^	*Lolium multiflorum*†	**-**
^(33), (4)^	*Lolium perenne*	*+*	^(38)^	*Phalaris arundinacea*†	**-**
^(30), (15)^	*Lolium persicum*	+	^(30)^	*Phalaris minor*	**-**
^(4), (37)^	*Lolium remotum*	+	^(38)^	*Phleum pratense*†	**-**
^(31), T^	*Lolium sp.*	+	^(33)^	*Poa pratensis*†	**-**
^(33), (4), (37)^	*Lolium temulentum*	+			

Positive (+) symbols indicate that WDV was detected at least once in the corresponding plant. Negative (-) signs showing plants that were tested but WDV could not be traced in the corresponding study. Crosses (†) mean that the plants were negative and positive as well in different studies. Names of the WDV reservoir species first published in this study are bold (shown in Fig. 1d, f). *Setaria pumila* (*) was identified first as *S. helvola* by the Flora Incognita smartphone application. T: this study

### WDV prevalence and genetic diversity

*WDV prevalence.* Among 250 field samples (91 leafhopper pools and 159 plants), 97 were WDV positive and 153 negative (Table 4). Of the positives, 74 were WDV-B and two were WDV-W (leafhoppers “K14” at site VIII and “CS18” at site XI). Three samples carried both strains (leafhopper pools from sites XI and XIII, and an *Arrhenatherum elatius* leaf from site XI). Eighteen positives did not react with any strainspecific primer and are provisionally treated as outgroup variants. Because of occasional false negatives (< 5%) persisted, each field sample was confirmed with at least two independent strain-specific sets that target different viral genes.

**Table 4:** Resolution of all collected samples by sample type and positivity. Pooled samples count as one. Samples taken from the in-house rearings were excluded from the table

				WDV positivity per all from sample type		WDV strain per positives from sample type			
				-	+	WDV-B	WDV-W	mixed	out-group
**all**			**250**	153 60.9%	**97** **39%**	74 76.2%	2 2%	3 3%	18 18.3%
sample type	leafhopper		**91**	74 80.4%	**17** **18.6%**	13 76.4%	2 11.1%	2 11.1%	0
	plant		**159**	79 49.7%	**80** **50.3%**	61 76.2%	0	1 1.2%	18 22.5%
	plant type	barley	**95**	34 35.78%	**61** **64.21%**	51 83.6%	0	0	10 16.4%
		wheat	**30**	20 66.6%	**10** **33.3%**	3 30%	0	0	7 70%
		other grass	**34**	25 73.5%	**9** **26.4%**	7 77.7%	0	1 11.1%	1 11.1%

All positives came from sites VIII, IX, XI and XIII (Fig. 2). Sites IX and XIII are only 2.53 km apart and sites VIII and XI 5.7 km apart—well within the flight range of *Psammotettix alienus*—so virus detections within each pair of sites may not be independent.

**Fig. 7:**
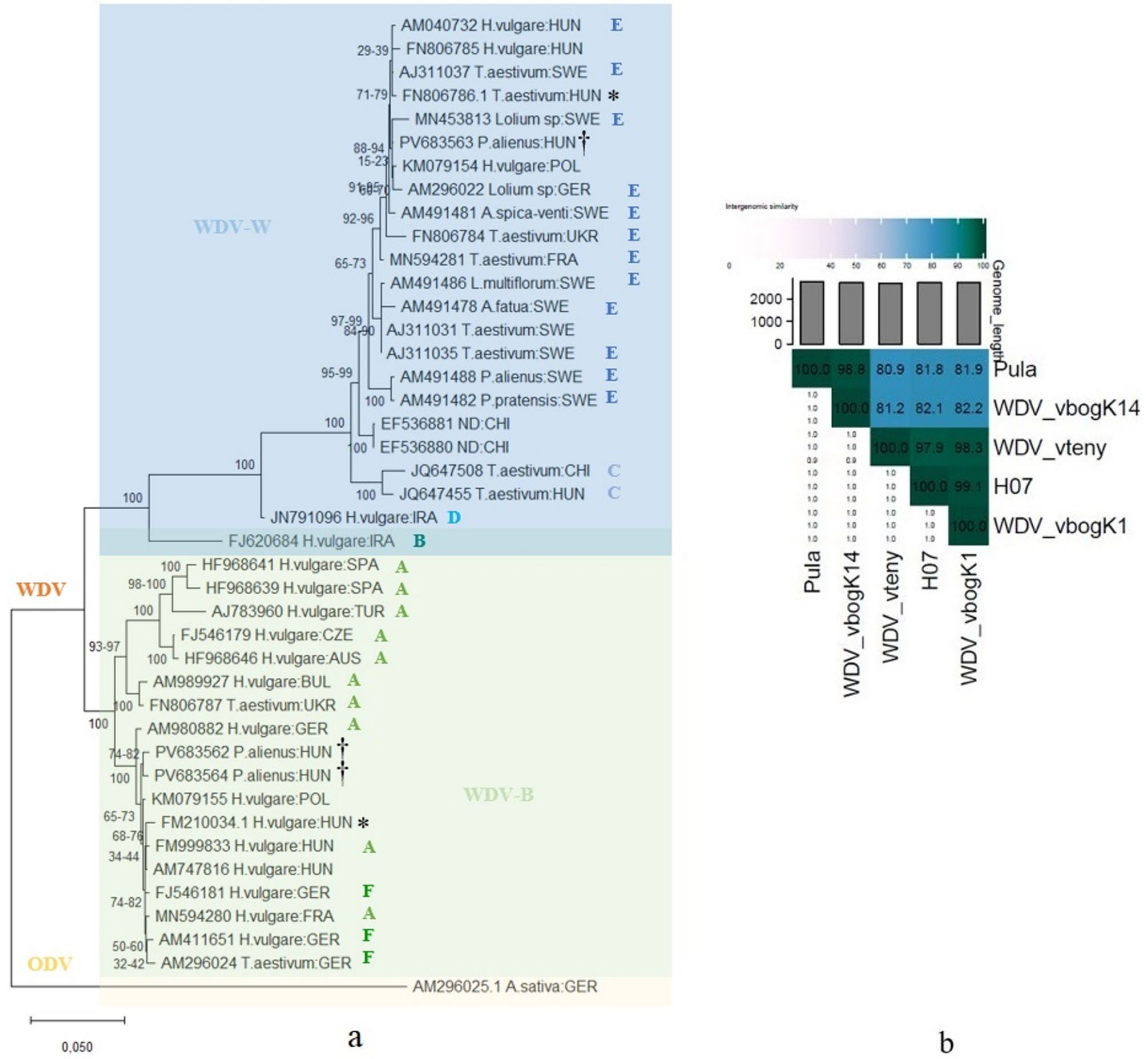
Sequence analysis of WDV genomes. (**a**) Phylogenetic tree of the compared WDV whole-genome sequences by MEGA12. The phylogeny was inferred using the maximum likelihood method (Note S3). Strains (WDV-B, WDV-W) and substrains (WDV/A-F) are indicated where known. An ODV sequence was used as an outgroup. Reference sequences H07 and pula are marked with asterisks, newly sequenced genomes with crosses. Accession numbers of the sequences correspond to the list in Table 1, isolation hosts and country abbreviations are indicated. (**b**) Comparison of the newly sequenced and reference isolate genomes. Heat map was made by the viridic platform

*Genomic characterisation of new WDV isolates*. BLASTn searches (Table S7) showed > 99% nucleotide identity within the barley strain (vbogK1, vteny, H07) and within the wheat strain (vbogK14, Pula), but only ≈ 85% identity between strains. Viridic pairwise distances were similar (Fig. 7b). Maximum-likelihood analysis placed vbogK1 and vteny firmly in the barley strain and vbogK14 in the wheat strain (Fig. 7a; Note S3). Complete genomes have been deposited in GenBank under accession numbers PV683562 (vbogK1), PV683563 (vbogK14) and PV683564 (vteny).

## Discussion

Earlier PCR assays for wheat dwarf virus were dispersed across laboratories, many yielding non-specific bands or failing when confronted with divergent isolates [27, 30]. By surveying many primer combination published to date, aligning thirty-eight complete genomes and bench-testing candidates on more than 150 field samples, we have replaced that fragmented information with a coherent toolkit— six generic and seven strain-specific primers that together span the V2, V1, C2 and C1 genes, coding for movement (MP), coat (CP), replication (Rep) and replication-associated (RepA) protein. The panel delivers clean amplification of both wheat and barley strains while tolerating known polymorphisms. As a proof of concept validation of the PCR toolkit, we had a survey of WDV infections across several regions in Hungary which unveiled a barley strain WDV dominated agricultural landscape in Central Hungary and revealed three novel grass reservoirs, demonstrating the ecological reach of the assay.

The validated genome-wide primer set for universal WDV diagnostics was created by collecting, retesting and - where necessary - upgrading published WDV primers [3, 6, 30, 32]. Additionally, we also created new primers to assemble a truly universal panel. During the tests, five primers—one generic and two for each strain—proved dysfunctional and were discarded, whereas four generic and four strain-specific oligonucleotides retained reliable in vitro performance. Three generic and two strain-specific primers were then minimally adjusted to accommodate all deposited genome variants, and five entirely new primers: cp1260-WDV_R, Rep1914-WDV-B_F, Rep1914WDVW_F, NFB17 H4 and NFB17 H5 were introduced so that each of the principal genome regions is now represented by at least two independent assays. Multilocus redundancy hedges against local mutations, while deliberate limitation of degeneracy preserves specificity, a balance that earlier singlelocus duplex PCRs could not reach [3, 32]. For routine users, a straightforward two-step workflow suffices: a generic screen with WDVrepDetDeg-For/Rev followed, when positive, by strain assignment with three independent assays: the Rep1914, the P1-P3 and/or the 1F-4R strain-specific pairs; Fig. 3; Table 2 list all workable combinations and qPCR adaptations. Unlike isothermal LAMP or rolling-circle protocols, which still await broad validation [24–26], the present PCR scheme can be implemented on any standard thermocycler. Moreover, the strain-specific pairs generate amplicons suitable for Sanger or next-generation sequencing, facilitating rapid phylogeographic analysis in the manner recently advocated by Wei, Liu [2] and Stanković, Zečević [22].

The discriminatory power of multilocus primer set was emphasised by the phylogenetic placement of three newly sequenced Hungarian isolates. Two barley strain genomes and one wheat strain genome shared > 99% identity within their respective strains but only ≈ 85% between strains, mirroring the ≈ 80% divergence that underpins current strain definitions [2, 8]. Interestingly, the Iranian isolate FJ620684– “substrain WDV/B” from barley [35]–, which shares near-equal nucleotide sequence similarity with both barley and wheat strains, clustered firmly with barley strain genomes once full-length alignments were used [9], illustrating how reference bias can mislead topology [36]. This ambiguity mirrors broader patterns in WDV evolution, where a supposed host-driven adaptation creates divergent strains (WDV-barley and WDV-wheat), while occasional cross-host transmission and recombinations create hybrid patterns [2, 36]. These findings emphasize the need for standardized reference frameworks and multi-locus targeting to resolve complex evolutionary relationships in WDV populations [31].

The virtual impossibility of field diagnosis of WDV by symptoms underlines the need for reliable molecular diagnosis. Classic dwarfing, chlorosis and spike sterility appeared in barley at Vértesboglár, yet infected yellow foxtail (*Setaria pumila*) and volunteer proso millet (*Panicum miliaceum*) showed only faint streaks. Winter wheat at Bakonykúti showed only yellowing in October, however, later observation in February found that WDV-B virtually eliminated wheat on that field. Conversely, every stunted plant at Csúza-Vörösmart proved virus-free. Such inconsistencies mirror experimental work showing that both host genotype and temperature modulate symptom expression [33, 37, 38]; at 35 °C symptoms may even remit despite ongoing replication [39]. The Csúza false positives reinforce the point: only molecular assays provide dependable surveillance, for which the primer panel offers a reliable solution.

The utility of the primer panel was proven in extensive field work carried out several regions in Hungary. Applying the primer set to 250 environmental samples from thirteen Hungarian sites yielded 97 positives, of which 76% belonged unambiguously to the barley strain (Table 4), uncovering a surprising barley strain dominance in the Middle-Transdanubian landscape, where winter wheat is the dominant cereal crop. The asymmetry was most dramatic at Vértesboglár, where a spring epidemic on self-sown barley peaked in the beginning of May: twenty-seven of twenty-eight randomly chosen or symptomatic plants and the majority of *Psammotettix alienus* specimens carried WDV-B. By late summer the same strain permeated wild grasses, emphasising its capacity to breach nominal host barriers when vectors disperse. At Bakonykúti, the barley strain even displaced its wheat counterpart in the standing wheat crop, with three of seven infected plants amplifying only WDV-B markers. This also contradicted the pattern deduced by Abt, Souquet [3], who posited that the presence of WDV-W isolates in barley and WDV-B isolates in wheat is likely to be exiguous in fields.

The detection of mixed or hybrid infections during the field surveys added another layer. A single *A. elatius* leaf at Csákvár yielded both barley- and wheat-specific amplicons yet none of them at the same primer site, hinting at a recombinant variant of the virus. Laboratory work shows that vectors carrying both strains can trigger cross-helper trans-complementation in non-preferred hosts [3]. Our field detections of barley strain take-over in wheat thus raise the possibility that such molecular cooperation operates in nature—an idea reinforced by the asymmetric BYDV–WDV coinfection dynamics recently reported in wheat [40]. Meanwhile all outgroup samples were negative to all selective primer combinations, suggesting that a similar molecular process might took place. However, artifact observation due to vector-derived surface contamination by the other WDV strain via salivary sheaths [41] or honeydew contamination [42] without an actual systemic infection of the examined plants should not be excluded from consideration as a possibility.

The field surveys also recovered three novel hosts plants, where WDV infection was confirmed by PCR. These expand the known reservoir spectrum. *Arrhenatherum elatius* and *S. pumila* were positive in October when cereals had been harvested, suggesting a bridge role through the summer fallow, while volunteer *P. miliaceum*—previously only ELISApositive [43]—harboured WDVB at several sites, corroborating its reputation as a recalcitrant weed and virus deposit [44–46]. These additions raise the global list of natural hosts to forty-five species [4, 15, 47]. Sampling at Isztimér further revealed that virus titres peaked inside a millet-dominated grassy island and decayed towards cultivated ground, implicating such refuges as inoculum cores much as earlier Swedish surveys implicated ryegrass margins [4, 15, 32, 47].

Landscape elements that enrich biodiversity can simultaneously sustain WDV. Reduced tillage and low herbicide regimes promote volunteer crops and wild grasses, raising virus prevalence by autumn [30, 43], yet those same margins shelter predator guilds —especially spiders [48, 49]— that suppress *P. alienus* [29, 50]. It is important to investigate the balance between intensive and reduced agricultural technology, for both seems to induce positive and negative effects on WDV spread and perseverance. Only strain-resolved, quantitative monitoring can balance these opposing forces.

Even though an elaborate and well-designed PCR could be considered a reliable detection method, this research encourages experts in this field to support their results with orthogonal tests (e.g. ELISA). Our research also develops LAMP technique as a quick on-the-spot application and an alternative confirmatory method for strain-specific WDV detection, detailed in our different study (under review). Our future prospect is to use LAMP, PCR and ELISA test simultaneously on the collected samples.

## Conclusions

Our study by identifying recommended primer sets, hopefully contributes to the better understanding of WDV epidemics. Apart from the biological foundation, it is important to monitor real world scenarios not only for the advancement of our scientific understanding, but for practical prevention purposes. Because the new primer panel discriminates strains and, in qPCR mode, quantifies load, it is ideally suited for adaptive management: thresholds could be set for targeted mowing of millet-dominated refuges or for timed insecticide treatments that spare beneficial arthropods.

Beyond WDV, the design logic—multilocus redundancy combined with minimal degeneracy—offers a blueprint for diagnostics of other leafhopper or aphid-borne plant viruses, such as oat dwarf and barley yellow dwarf viruses, where heterogeneous reservoirs similarly complicate control [9, 11, 51]. Our primer architecture coupled with existing methodologies would bring field-level genotyping within reach of crop consultants and even growers and help to find the right management practices in the case of plant diseases other than WDV.

## Electronic supplementary material

Below is the link to the electronic supplementary material.

Supplementary Material 1

## Data Availability

Data is provided within the manuscript or supplementary information files.

## Abbreviations

BDV: barley dwarf virus
CP: coat protein
DAS-ELISA: double antibody sandwich enzyme-linked immunosorbent assay
ETS: external transcribed spacer
ITS: internal transcribed spacer
LAMP: loop-mediated isothermal amplification
LIR: long intergenic region
matK: maturase K
MP: movement protein
ODV: oat dwarf virus
PCR: polymerase chain reaction
qPCR: quantitative polymerase chain reaction
RCA: rolling-circle amplification
SIR: short intergenic region
SNP: single nucleotide polymorphism
WDV: wheat dwarf virus
WDV-B: wheat dwarf virus, barley strain
WDV-W: wheat dwarf virus, wheat strain
