# Supplementary material for: Widely-based full-genome analyses enable development of universal and strain-specific PCR toolkit for wheat dwarf virus detection, revealing new alternative hosts and challenging strain-host specificity
Source: Plant Methods. 2025 Jul 21;21:100. doi: 10.1186/s13007-025-01420-6 (PMC12278524; doi:10.1186/s13007-025-01420-6)
Supplement: Supplementary file 1 — Supplementary Material 1 [file 13007_2025_1420_MOESM1_ESM.docx]

Table S1. Timeline and locations of the WDV sampling in year 2023 and 2024. Roman numerals of the sampling sites correspond with Fig. 2. GPS coordinates of the sites are listed in Table S3. Each sampling event is denoted by a cell. Event numbering corresponds to the list in Table S2. Gray background and bold letter font indicates positivity for WDV among the samples.

|  |  | **2023** | | | | | | | | | **2024** | | | | | | | |
| --- | --- | --- | --- | --- | --- | --- | --- | --- | --- | --- | --- | --- | --- | --- | --- | --- | --- | --- |
|  | sampling site | 10^th^ Apr. | 21^st^ Apr. | 9^th^ May | 30^th^ June | 17^th^ July | 13^th^ Aug. | 27^th^ Sept. | 3^rd^ Oct. | 16^th^ Nov. | 21^st^ February | 3^rd^ May | 9^th^ May | 23^rd^ May | 25^th^ July | 23^rd^ Sept. | 9^th^ Oct. | 26^th^ Oct. |
| **Ia** | Martonvásár, Rozsdakert |  |  |  | 7 |  |  |  |  | 13 | 15 |  |  |  |  | 17 |  |  |
| **Ib** | Martonvásár, Búza utca |  |  |  |  |  |  |  |  | 14 | 16 |  |  |  |  |  |  |  |
| **II** | Környe |  | 4 | 5 | 6 |  |  |  |  |  |  |  |  |  |  |  |  |  |
| **IIIa** | Csúza, kert | 1 |  |  |  |  |  |  |  |  |  |  |  |  |  |  |  |  |
| **IIIb** | Csúza, Kisvölgy | 2 |  |  |  |  |  |  |  |  |  |  |  |  |  |  |  |  |
| **IIIc** | Vörösmart,  Bellye borászat | 3 |  |  |  |  |  |  |  |  |  |  |  |  |  |  |  |  |
| **IV** | Érd, Elvira-major |  |  |  |  |  | 9 |  |  |  |  |  |  |  |  |  |  |  |
| **V** | Budakeszi, kert |  |  |  |  |  |  | 10 |  |  |  |  |  |  |  |  |  |  |
| **VI** | Budapest, Óbuda |  |  |  |  | 8 |  |  |  |  |  |  |  |  |  |  |  |  |
| **VII** | Budapest,  Júlia-major |  |  |  |  |  |  | 11 | 12 |  |  |  |  |  |  |  |  |  |
| **VIII** | Vértesboglár, Félszermajor |  |  |  |  |  |  |  |  |  |  | **18** | **19** | **20** | 21 | **22** |  | **23** |
| **IX** | Isztimér |  |  |  |  |  |  |  |  |  |  |  |  |  | **24** |  | 25 |  |
| **Xa** | Lovasberény |  |  |  |  |  |  |  |  |  |  |  |  |  |  |  | 26 |  |
| **Xb** | Lovasberény |  |  |  |  |  |  |  |  |  |  |  |  |  |  |  | 27 |  |
| **XI** | Csákvár |  |  |  |  |  |  |  |  |  |  |  |  |  |  |  | **28** | **29** |
| **XII** | Söréd |  |  |  |  |  |  |  |  |  |  |  |  |  |  |  | 30 | 31 |
| **XIII** | Bakonykúti |  |  |  |  |  |  |  |  |  |  |  |  |  |  |  | **32** | **33** |

Table S2. Detailed timeline and conditions of the different sampling events. Sample positivity for WDV is also listed. For a detailed description, see Note S1 and S2.

| **site** |  | **properties of the sampling location** | **sample type and amount** | **sample positivity for WDV** |
| --- | --- | --- | --- | --- |
| III Csúza, Vörösmart | **1** | garden, empty field margin | grasses (4 samples) | negative |
|  | **2** | spring barley field (Fig. S11) | barley (2 samples) | negative |
|  | **3** | spring wheat field (Fig. S11) | wheat (4 samples) | negative |
| II Környe | **4** | spring barley field | barley (20 samples) | negative |
|  | **5** |  | barley (1 pool) | negative |
|  | **6** |  | *P. alienus* to stock ‘MV’* | *positive***, WDV-B |
| Ia Marton RK | **7** | wheat field |  |  |
| VI BP, Óbuda | **8** | 3^rd^ floor flat, barley in the window | *P. alienus* (1 specimen) | negative |
| IV Érd, Elvira | **9** | empty grain field | *P. alienus* (2 pools) | negative |
| V Budakeszi | **10** | garden | *P. alienus* (pool) | negative |
| VII BP, Júlia-major | **11** | fallow with weeds (*Setaria* sp*., E. crus-galli*) | *P. alienus* (pool) | negative |
|  | **12** |  | *P. alienus* (pool) | negative |
| Ia Marton, RK | **13** | winter wheat field | *P. alienus* to rearing, then pool‡ | negative |
| Ib Marton, Búza u. | **14** | winter wheat field | *P. alienus* to rearing, then pool‡ | negative |
| Ia Marton, RK | **15** | winter wheat field | wheat (pool) | negative |
| Ib Marton, Búza u. | **16** | winter wheat field | wheat (pool) | negative |
| Ia Marton, RK | **17** | fallow with weeds | grass (pool)  *P. alienus* to stock ‘MartonM’* | negative  *virus-free*** |
| VIII Vértesboglár | **18** | reposed field  (Fig.S1) | 8 potted barley  8 sampled barley  *P. alienus* (16 samples) ⁂ | 1/8 positive  5/8 positive, all WDV-B  13/16 **positive**: 12 WDV-B, 1 WDV-W |
|  | **19** | self-sown barley with growing *P. tanacetifolia* and *P. miliaceum* (Fig. S2) | barley (2x28 linear samples)  (Fig. 1a) | 55/56 **positive**: 45 WDV-B, 10 outgroup*** |
|  | **20** | *P. tanacetifolia* is dominant  (Fig. S3) | *Cynodon dactylon*  *B. tectorum, A. spica-venti*, *P. miliaceum*, *B. hordeaceus*, *F. pratensis* | **positive**, WDV-B  not tested, for potting and seed propagation |
|  | **21** | *P. miliaceum* is dominant  (Fig. S4) | *P. miliaceum* (10 samples)  *P. alienus* to stock ‘Vbog’* | all negative  *positive**,* WDV-B |
|  | **22** | fallow with weeds  (Fig. S5) | *S. pumila, P. miliaceum, C. dactylon* (1-1-1 samples) (Fig. 1d, e)  *S. pumila* and *P. miliaceum* potted | **all 3 positive**, WDV-B  **both positive**, WDV-B |
|  | **23** | empty, freshly plowed field, w/o margin grasses (Fig. S6) | 1 grass sample from neighboring greensward (*Lolium* sp.) | **positive**, outgroup*** |
| IX Isztimér | **24** | arrish (stubble-field)  (Fig. S7) | *P. miliaceum* (1 sample)  *P. alienus* to stock ‘Iszt’* | **positive**, WDV-B  *positive**,* WDV-B |
|  | **25** | fallow with weeds | *S. pumila, P. miliaceum,* grass (1-1-1 samples) | all negative |
| Xa Lovasberény | **26** | self-sown wheat table  field margin | *P. alienus* (pool)*,* wheat (1 sample)  grasses (1 sample) | negative, negative  negative |
| Xb Lovasberény | **27** | self-sown wheat table  field margin | wheat (1 sample)  *P. miliaceum* (1 sample) | negative  negative |
| XI Csákvár | **28** | fallow with weeds  field with self-sown grass  margin | *P. alienus* (pool), *Setaria* sp. (1 sample)  *P. alienus* (pool), *Triticum* sp. (1 sample)  grasses (1 sample) | negative, negative  **positive** (WDV-B and W), negative  negative |
|  | **29** | freshly tilled field, field margin  (Fig. S8) | *P. alienus* (39 samples)  *A. elatius* (2 samples) (Fig. 1f)  *Bromus* sp. (1 sample)  *P. alienus* to stock ‘Csákvár’* | 2/39 **positive**: one WDV-B, one WDV-W  1/2 **positive**: mixed WDV-B, WDV-W  negative  *positive***, WDV-B |
| XII Söréd | **30** | wheat field | *P. alienus* (pool)*, Zea mays,*  wheat (1-1 samples) | all negative |
|  | **31** | wheat field | *P. alienus* (pool)  *P. alienus* to stock ‘Söréd’* | negative  *virus-free*** |
| XIII Bakonykúti | **32** | winter wheat field | *P. alienus* pool, wheat pool | **positive** (WDV-B and W), negative |
|  | **33** | winter wheat field  (Fig. S10) | *P. alienus* (22 samples)  wheat linear (20 samples) (Fig. 1g) | all negative  10/20 **positive:** 3 WDV-B, 7 outgroup*** |

* Source of the in-house rearings.

** Leafhopper or plant samples were taken from the rearings occasionally. These samples do not count in Table 5.

*** Generic WDV positive samples were not further positive neither with WDV-B, nor with WDV-W primers.

‡ Rearing perished within a few weeks.

**⁂** Two leafhopper samples (K1 and K14, WDV-B and WDV-W, respectively) were sequenced.

Table S3. GPS coordinates of the sampling sites.

|  | sampling site | GPS coordinates |
| --- | --- | --- |
| **Ia** | Martonvásár, Rozsdakert | 47.318360, 18.775281 |
| **Ib** | Martonvásár, Búza utca | 47.328521, 18.787784 |
| **II** | Környe | 47.534779, 18.341149 |
| **IIIa** | Csúza, kert | 45.781044, 18.779023 |
| **IIIb** | Csúza, Kisvölgy | 45.791583, 18.769672 |
| **IIIc** | Vörösmart, Bellye borászat | 45.800435, 18.799387 |
| **IV** | Érd, Elvira-major | 47.353308, 18.857969 |
| **V** | Budakeszi, kert | ??? |
| **VI** | Budapest, Óbuda | 47.546499, 19.032262 |
| **VII** | Budapest, Júlia-major | 47.548314, 18.933918 |
| **VIII** | Vértesboglár, Félszermajor | 47.405780, 18.556647 |
| **IX** | Isztimér | 47.271825, 18.194704 |
| **Xa** | Lovasberény | 47.343613, 18.555866 |
| **Xb** | Lovasberény | 47.337665, 18.547626 |
| **XI** | Csákvár | 47.369127, 18.504102 |
| **XII** | Söréd | 47.324427, 18.290274 |
| **XIII** | Bakonykúti | 47.249799, 18.187759 |

Table S4. Primers used in this study for plant and leafhopper identification.

| **code** | **PCR cycles*** | **primer name** | **5’-3’sequence** | **target** | **source** |
| --- | --- | --- | --- | --- | --- |
|  | B | 200bp_3H_Fw | CATGTGATCTCCCACCGGCT | barley genomic | MBK (Ali et al., 2024) |
|  | B | 200bp_3H_Rv | GGAGCGACGTTGAGAACTGC | barley genomic | MBK (Ali et al., 2024) |
| 37 | B | 400bp_F6927_7A | TGGCGGCCATTAGACTAGCG | wheat A genome | MBK (Ali et al., 2024) |
| 38 | B | 400bp_R6927_7A | GGCGTCTCAGGTGTTGGACT | wheat A genome | MBK (Ali et al., 2024) |
| 41 | B | 400bp_F7805_7D | AGCGTCGCGTTCGTTCCATG | wheat D genome | MBK (Ali et al., 2024) |
| 42 | B | 400bp_R7805_7D | CAAGCGTTCCACTCCGGTCT | wheat D genome | MBK (Ali et al., 2024) |
|  | B | Psam268F | ACCACCATCTATCACCCTACT | *P. alienus* COI | (Fulop et al., 2019) |
|  | B | Psam435R | GCCCCAAGGATTGAGGAGAT | *P. alienus* COI | (Fulop et al., 2019) |
| 125 | C | ITS1-F | GGAAGGAGAAGTCGTAACAAGG | *Poaceae* ITS-1 | (Omelchenko et al., 2022) |
| 126 | C | ITS1-R | AGATATCCGTTGCCGAGAGT | *Poaceae* ITS-1 | (Omelchenko et al., 2022) |
| 131 | C | RETS-B1F | WGKCTATCRACGTGCATGAG | *Pooideae* 3’-ETS | (Alonso et al., 2014) |
| 132 | C | RETS-B2F | TATCAACGTGCATGAGTAGC | *Pooideae* 3’-ETS | (Alonso et al., 2014) |
| 136 | C | 18SR | AGACAAGCATATGACTACTGGCAGG | ETS | (Starr et al., 2003) |
| 43 | C | matK-xf_Fw | TAATTTACGATCAATTCATTC | chloroplast *mat*K | (Kuzmina et al., 2017) |
| 44 | C | MatK-MALP_Rev | ACAAGAAAGTCGAAGTAT | chloroplast *mat*K | (Kuzmina et al., 2017) |

*PCR CYCLES:

B: 15 min 95°C, 35 cycles of 30 s 95°C, 30 s 55°C, 1 min 72°C, followed by 5 min 72°C.

C: 15 min 95°C, 35 cycles of 30 s 95°C, 30 s 42°C, 40 s 72°C, followed by 10 min 72°C.

## Note S1: Sampling conditions

Site-group IIIa-c (Csúza and Vörösmart) was a distal sampling site in county Baranja, Croatia. Grass, wheat and barley were collected for direct WDV testing (event 1-3).

At site Ia (Martonvásár, Rozsdakert) several samplings were performed at different times (event 7, 13, 15, 17): plants and animals were collected and were tested for WDV directly and gathered to start a parallel rearing.

At site VIII (Vértesboglár, Félszermajor), also several samples were taken on different occasions on a fallow land, a former barley field (Fig S1). In May 2024, the field was secondary sowed with purple tansy (*Phacelia tanacetifolia*) and proso millet (*Panicum miliaceum*) with a significant presence of self-sown barley (Fig. S2, S3). On 3^rd^ May (event 18), animals were collected for direct testing (N=16). For plant sampling (9^th^ May, event 19, Fig. S2), a linear method was applied: 2x14 random plant samples (barley) were collected from the field across a line, with 20 steps in between the sampling points. Also, 2x14 symptomatic barley plants (yellow leaves, heavy streaking, dwarfing, Fig. 1a) were collected at the same time. On 23^rd^ May (event 20, Fig. S3), different grasses were sampled, mostly from the field margin: downy brome (*Bromus tectorum*), common windgrass (*Apera spica-venti*), proso millet (*P. miliaceum*), soft brome (*Bromus hordeaceus*), meadow Fescue-grass (*Festuca pratensis*) for potting, and Bermuda grass (*Cynodon dactylon*) for direct virus detection. In July (event 21, Fig. S4), *P. miliaceum* was dominant on the field, which was sampled for direct virus detection. Animals were collected for parallel rearing (‘Vbog’); on the same day, from site IX (Isztimér), animals were also collected for the same purpose (rearing ‘Iszt’, event 24, Fig. S7). At the end of September (event 22, Fig. S5), the field had no dominant plant species and no leafhoppers were found. Bermuda grass (*C. dacytylon*), proso millet (*P. miliaceum*) (Fig. 1e) and yellow foxtail (*Setaria pumila*) (Fig. 1d) were taken for direct virus testing, one-one symptomatic specimen from the latter two were also potted (Fig. S12). In October (event 23, Fig. S6) the whole field was freshly plowed without leaving any field margin. A grass sample (*Lolium* sp.) was taken from the neighboring green meadow.

At sites XI (Csákvár) and XIII (Bakonykúti), two samplings were performed at various times (beginning and end of October). At the former occasion (event 28 and 32), only pooled plant and leafhopper samples were taken (1-1 at both places), at the latter occasion (event 29 and 33; Fig. S8 and S10), 39 and 22 individual leafhoppers were captured, respectively; 3 grass samples at Csákvár (2 *Arrhenatherum elatius* and 1 *Bromus* sp.) (Fig. 1f), and 20 wheat samples (Fig. 1g) at Bakonykúti on a linear gradient. This was performed by advancing in a diagonal line across the wheat field and collecting samples with 10 steps in between the 20 sampling points (Fig. S10).

## Note S2: WDV spatio‑temporal dynamics and host plants at three hot-spot sites

***Site VIII (Vértesboglár)***

*Early May.* On 3 May (event 18) 16 leafhoppers were collected: 12 carried WDV‑B and one (isolate K14) WDV‑W. Six days later, on 9 May (event 19), linear sampling across the fallow dominated by self‑sown barley revealed an epidemic—28/28 symptomatic plants and 27/28 randomly chosen plants were WDV‑B‑positive (Fig. 1a; Fig. S2, Fig. 5). Leafhopper isolates K1 (WDV‑B) and K14 (WDV‑W) were sequenced.

*Late May.* A WDV‑B‑infected *Cynodon dactylon* plant was found on 23 May (event 20; Fig. S3). Transmission tests with virus‑free *Bromus tectorum* and *B. hordeaceus* remained negative after 1–2 weeks of leafhopper feeding (Fig. S13).

*Late July.* By 30 July (event 21) all ten *Panicum miliaceum* plants sampled on the fallow were virus‑free, yet leafhoppers collected that day to establish colonies (“vbog” and “iszt”) later tested as WDV‑B‑positive (Fig. S14). On the same date, at site IX (Isztimér, event 24; Fig. S7) the only positive was a small *P. miliaceum* patch in stubble; no positives were found there in October.

*Late September.* On 27 September (event 22; Fig. S5) heavily symptomatic *Setaria pumila*, *P. miliaceum* and *C. dactylon* were widespread, all infected with WDV‑B (Fig. 1d–e). Leafhoppers were absent, indicating these grasses had become a temporary virus reservoir.

*Early October.* When the site was inspected on 10 October (event 23; Fig. S6) the field had been freshly ploughed, removing the in‑situ reservoir; however, a single *Lolium* sp. plant in an adjacent meadow remained WDV‑positive.

***Site XI (Csákvár)***

– 9 October: a pooled leafhopper sample contained both strains, whereas all grass pools—from the empty field interior, the grassy margin and a neighbouring fallow—were virus‑negative.

– 26 October: only two of 39 individually tested leafhoppers were positive (CS21, WDV‑B; CS18, WDV‑W) despite dense vector activity along the margin (Fig. S8). Of three margin grasses, one *Arrhenatherum elatius* harboured a mixed infection (Fig. 1f); a second *A. elatius* and a *Bromus* sp. were negative.

***Site XIII (Bakonykúti)***

– 9 October: the pooled wheat sample was virus‑free, yet the leafhopper pool contained both strains. The wheat was 10–15 days old.

– 30 October: all 22 leafhoppers were virus‑negative, whereas ten of 20 wheat plants sampled along a transect were infected—mostly with WDV‑B or untypable out‑group variants (Fig. 1g). PCR and qPCR mapping (Fig. 6) showed highest virus titres around sampling points 4–5, secondary foci at 9 and 15–16, and negligible titres at 1–2, 10–13 and 18–19. Three wheat samples (5, 6, 9) reacted only with barley‑strain Rep primers, implying CP‑gene mutations or titres below CP‑assay sensitivity (Table S6).

## Note S3: Phylogenetic tree conditions in MEGA12

**Evolutionary analysis by the Maximum Likelihood method****.** The phylogeny was inferred using the Maximum Likelihood method and Tamura-Nei (1993) model of nucleotide substitutions and the tree with the highest log likelihood (-12 461.58) is shown. The percentage of replicate trees in which the associated taxa clustered together, where the number of replicates (110) was determined adaptively, is shown next to the branches. The initial tree for the heuristic search was selected by choosing the tree with the superior log-likelihood between a Neighbor-Joining (NJ) tree and a Maximum Parsimony (MP) tree. The NJ tree was generated using a matrix of pairwise distances computed using the p-distance. The MP tree had the shortest length among 10 MP tree searches, each performed with a randomly generated starting tree. The analytical procedure encompassed 42 coding nucleotide sequences using 1st, 2nd, 3rd, and non-coding positions with 2 734 positions in the final dataset. Evolutionary analyses were conducted in MEGA12 utilizing up to 3 parallel computing threads.


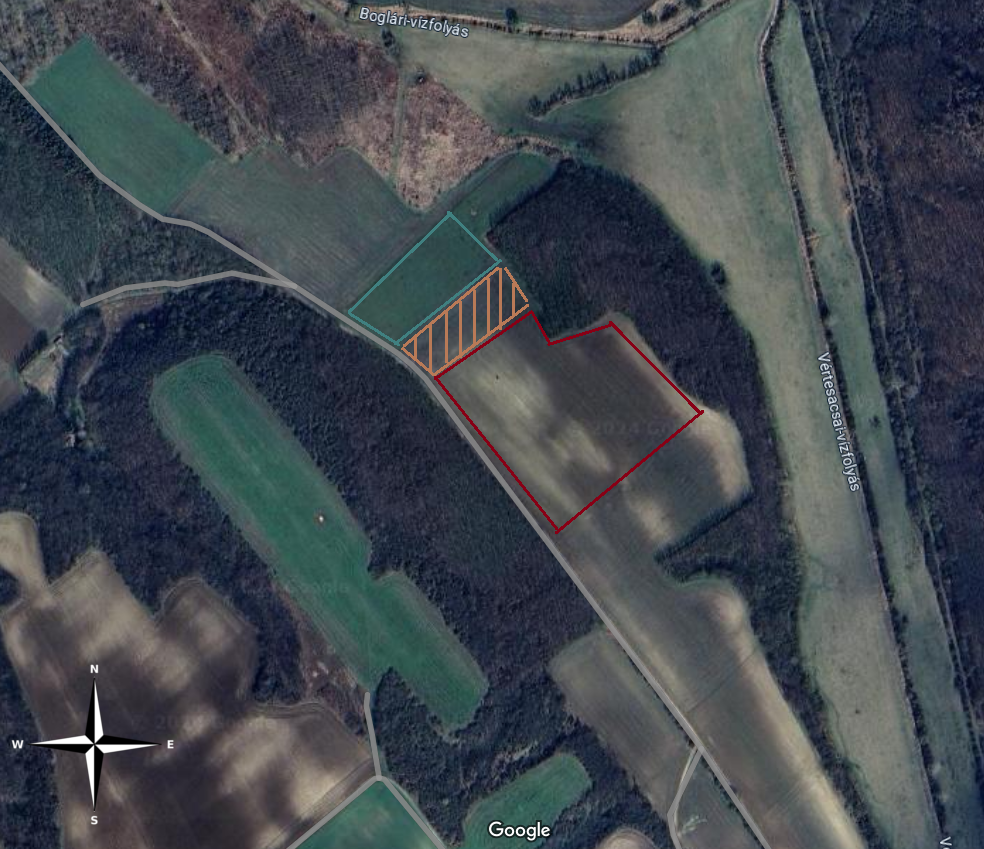


Figure S1. Aerial view of Site VIII Félszermajor, near Vértesboglár. The examined fallow field is framed by red lines, the striped rectangle shows a margin area with weeds, the green rectangle is a grassy meadow.
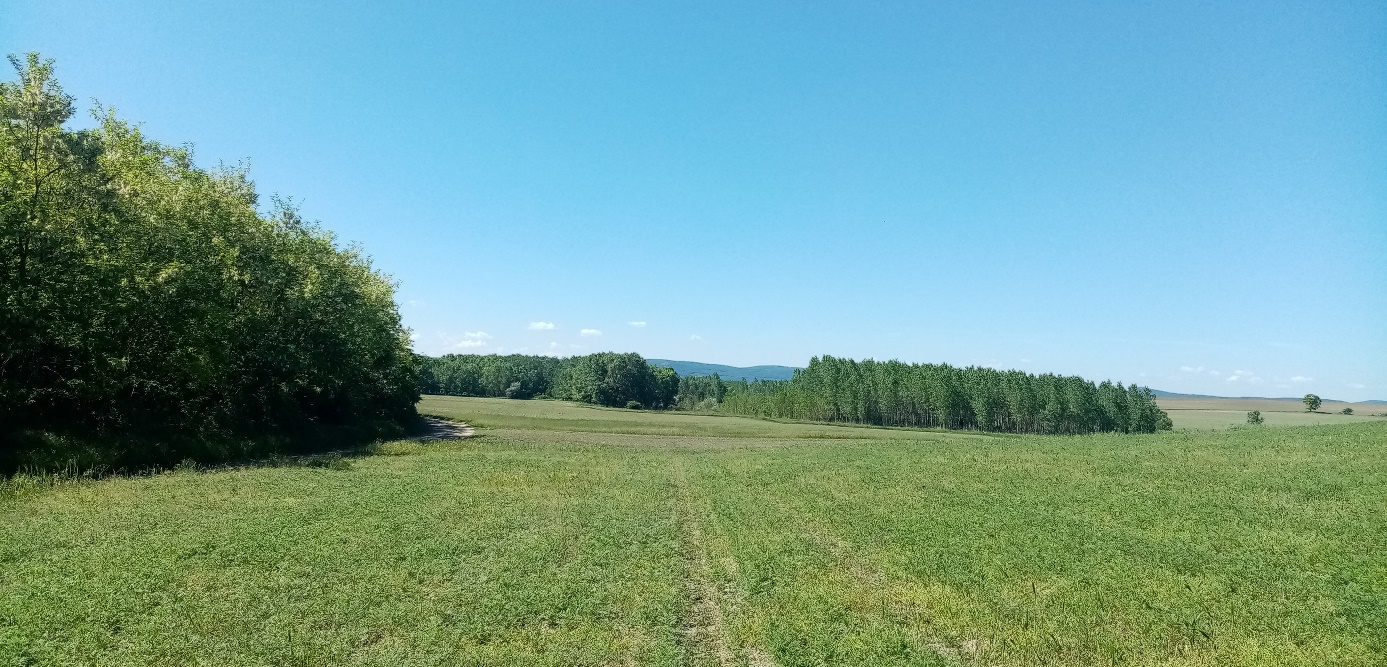

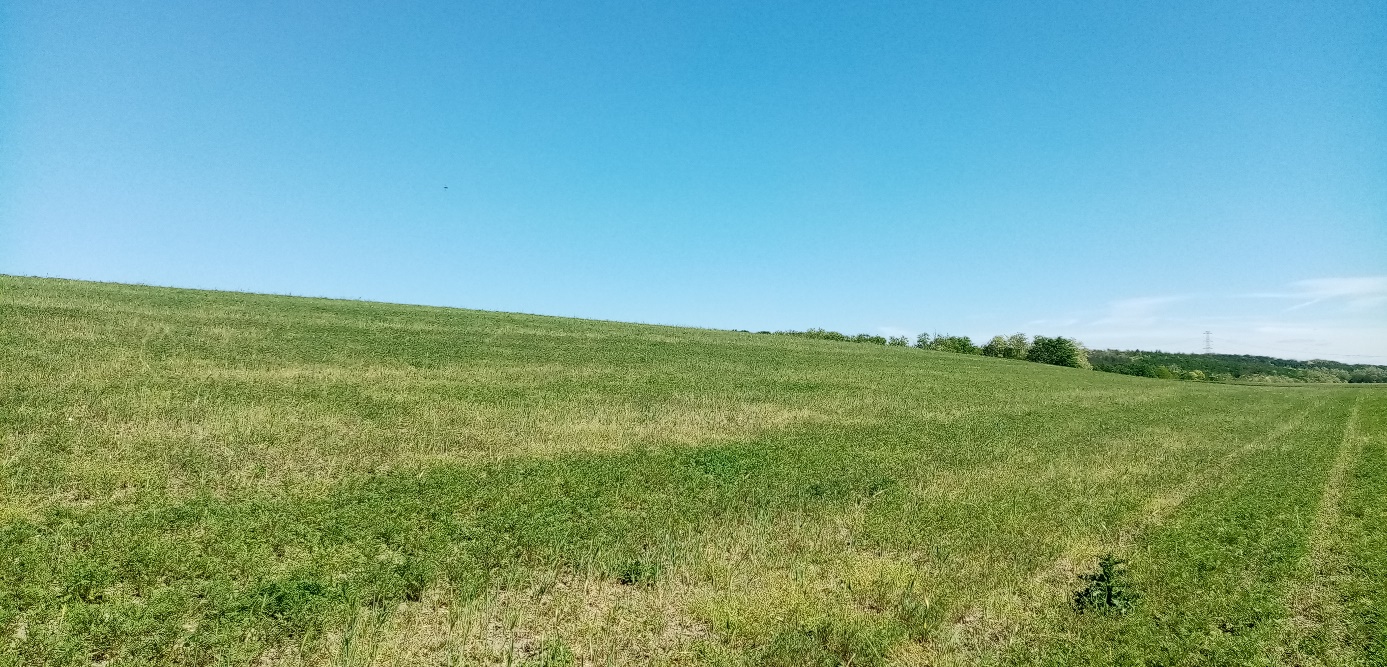


Figure S2. Site VIII on 9th May (event 19). *P. tanacetifolia* is growing, the field is full of self-sown, WDV-symptomatic barley.


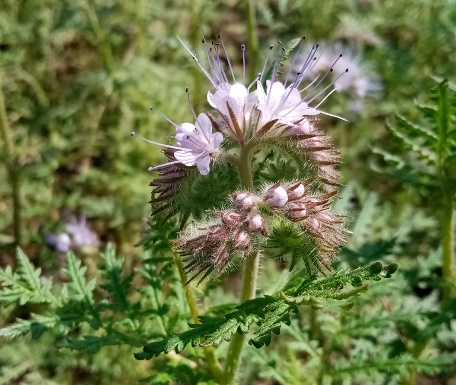

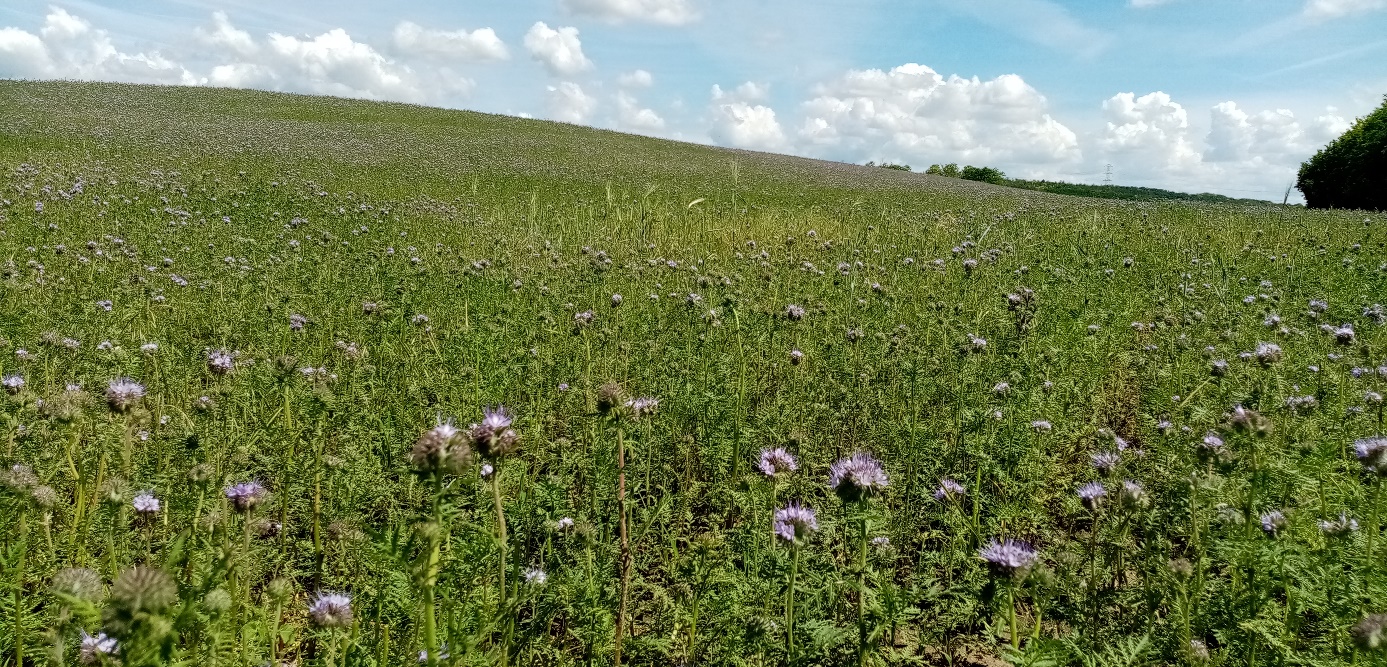

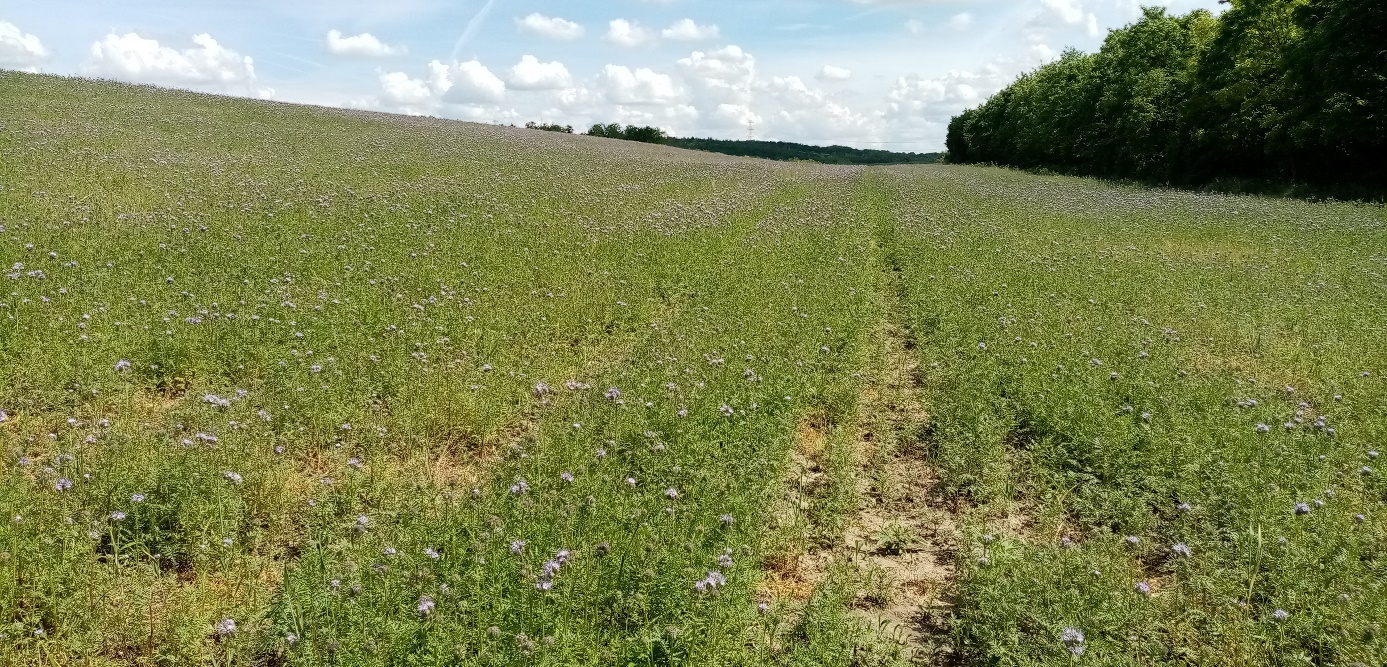


Figure S3. Site VIII on 23rd May (event 20). *P. tanacetifolia* is dominant.


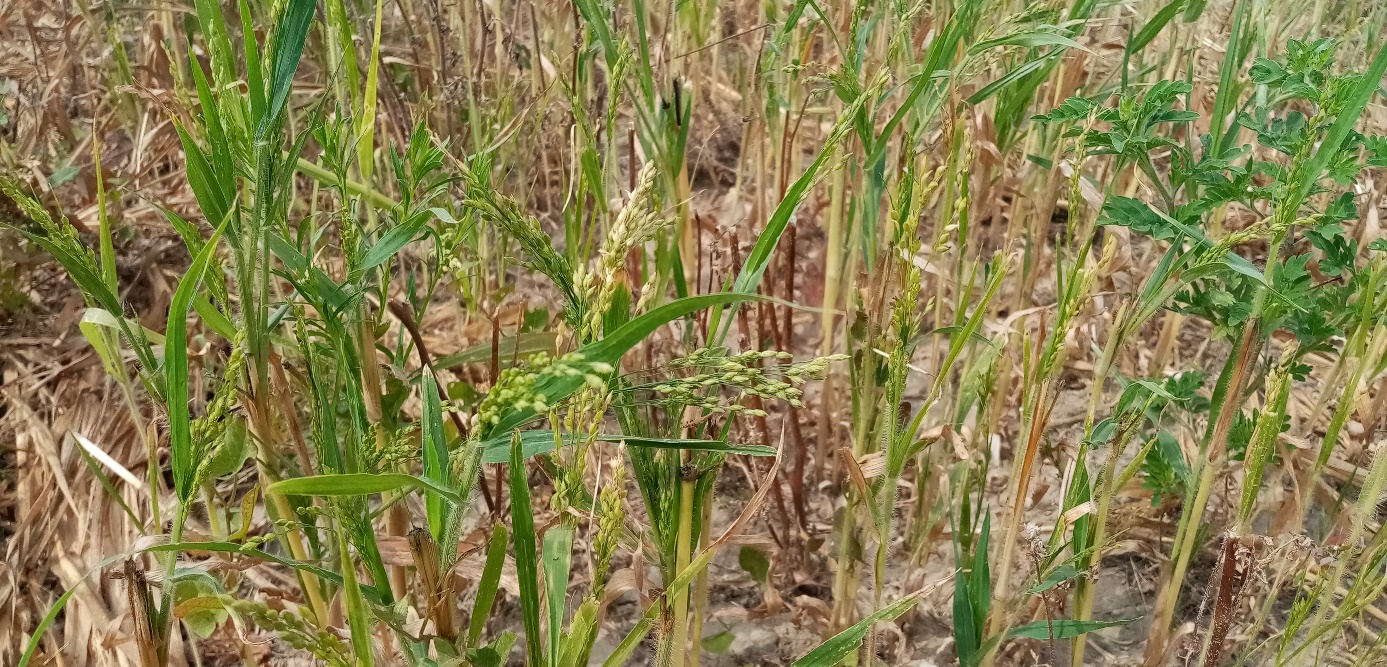

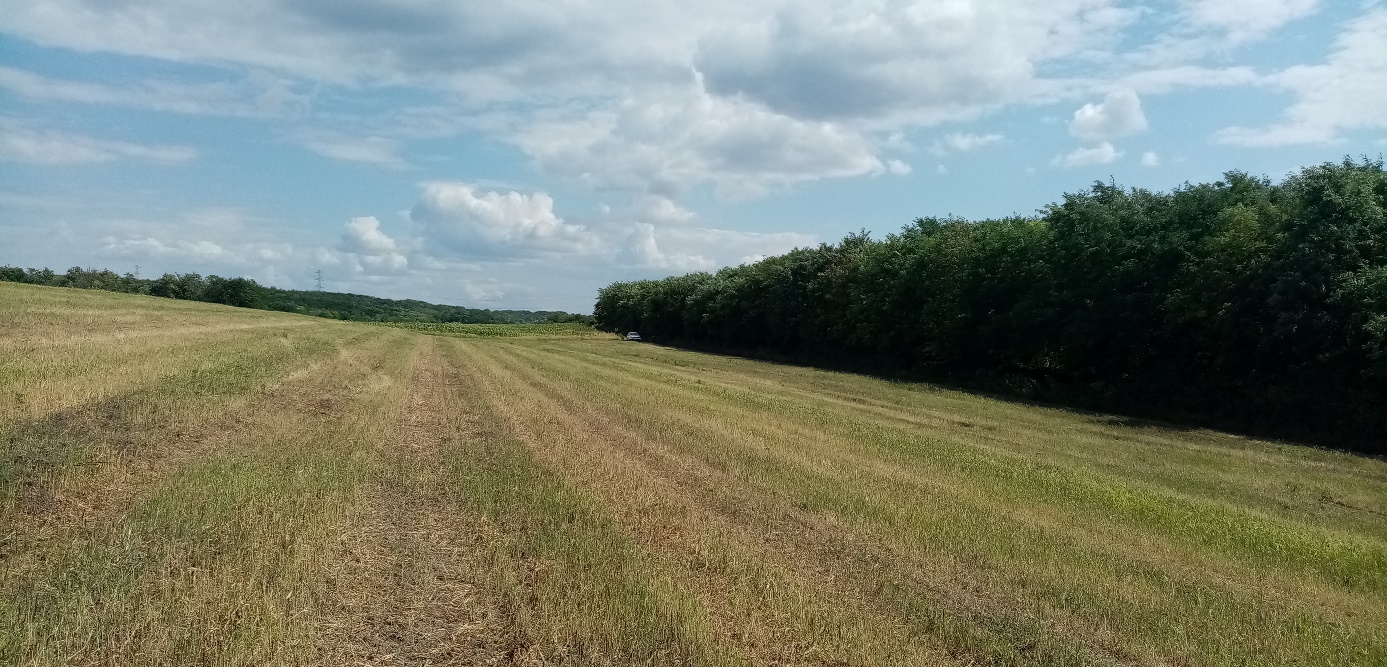


Figure S4. Site VIII on 25th July (event 21). *P. miliaceum* is dominant.


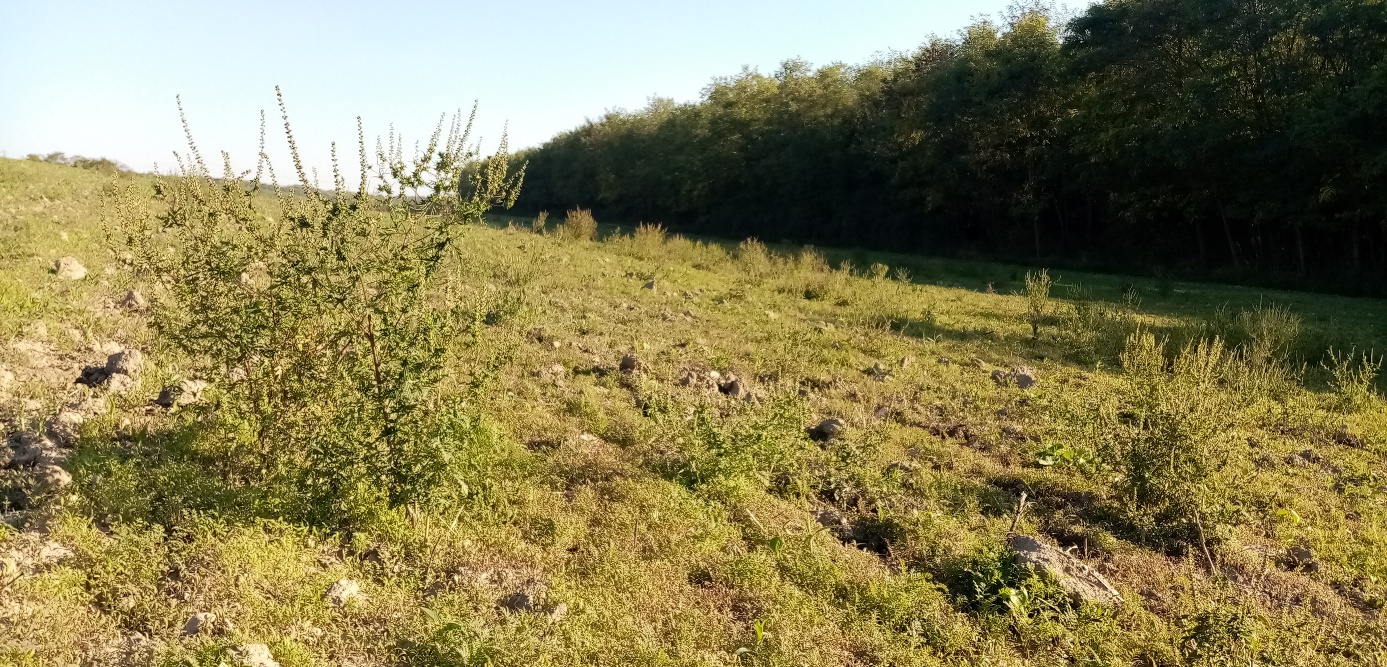

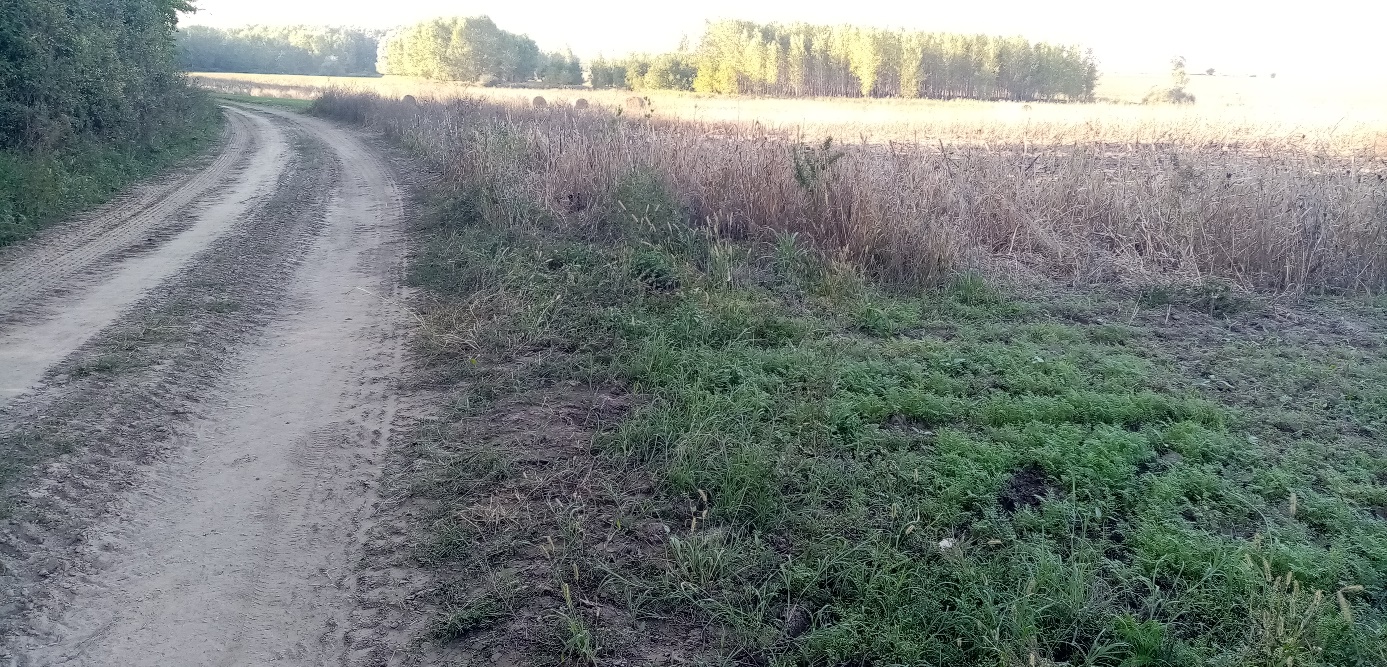


Figure S5. Site VIII on 23rd September (event 22). Red arrows show grasses that were sampled at the very corner of the field. The weedy area (brown arrow) is still intact between the field and the green meadow (pointed by the green arrow).


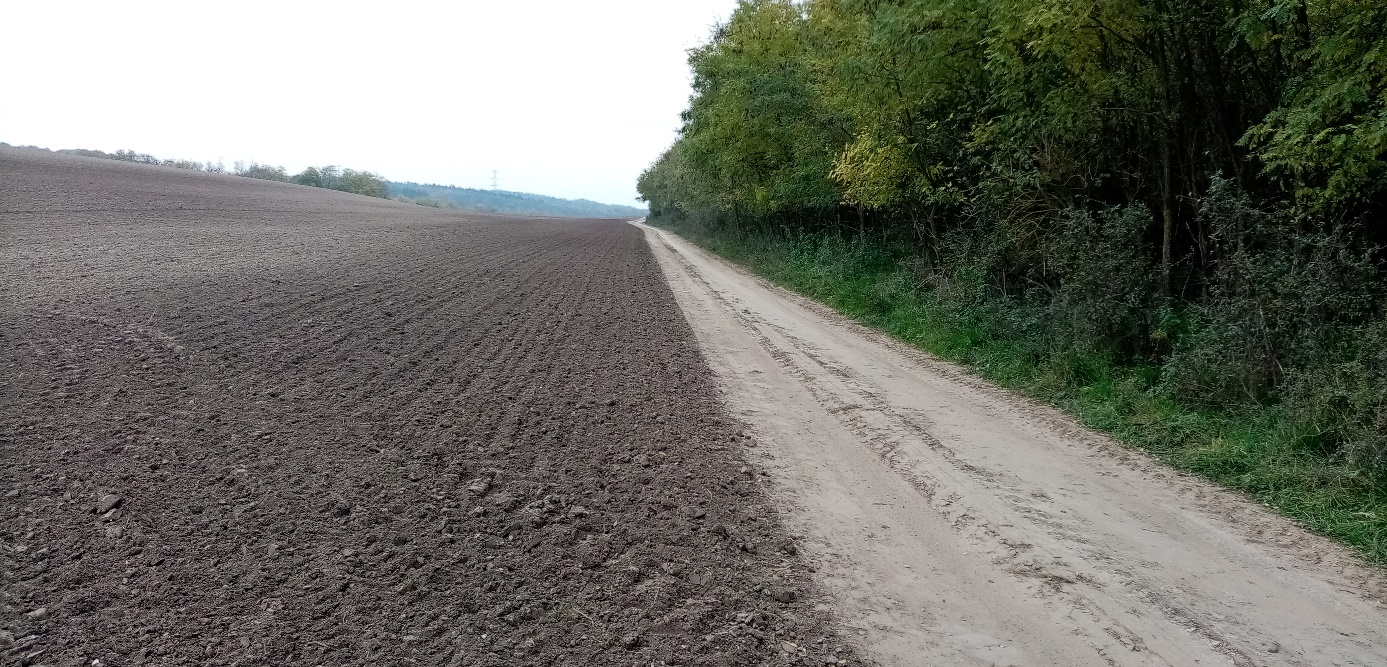

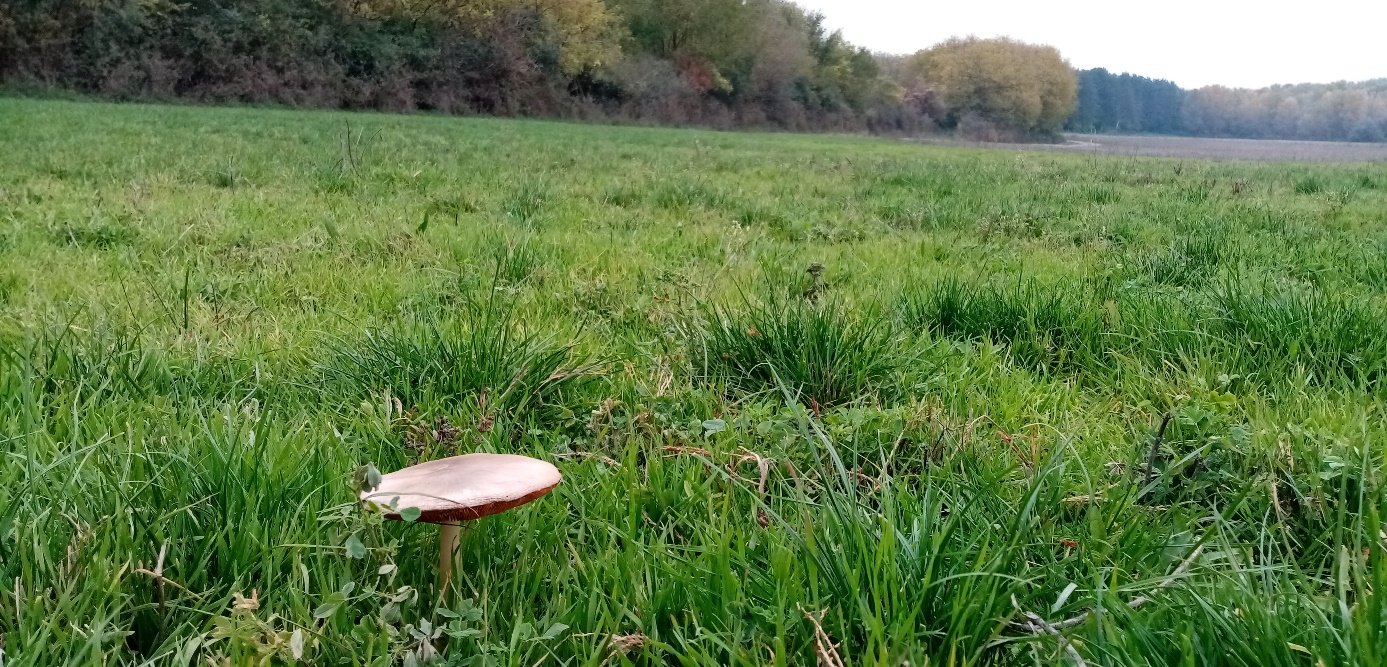


Figure S6. Site VIII on 26th October (event 23). The freshly-plown field without field margins. The weedy area was also plowed. The green meadow was intact.

Table S5. Testing of leafhopper samples K1, K13 and K14 (collected at event 18) for WDV specificity (up). K1 and K13 showed positivity for barley strain-specific primer-pair 87+91 (H amplicon), and further for primers 10+93 (N amplicon) and 93+31 (K amplicon). K14 showed positivity for the test with wheat strain-specific primer-pair 88+91 (I amplicon), and further for primers 9+91 (Q amplicon) and 93+90 (M2 amplicon). Samples H07 and Pula were used as controls for WDV-B and WDV-W, respectively. The bottom table shows the testing of WDV outgroup samples from the Random II batch. Samples were tested with generic primer pairs (30+94 and 93+96).

|  |  | | | K1 | | | K13 | | | K14 | | | H07 | | | Pula | |  |  |  |  |  |
| --- | --- | --- | --- | --- | --- | --- | --- | --- | --- | --- | --- | --- | --- | --- | --- | --- | --- | --- | --- | --- | --- | --- |
| WDV-W | **M2** | | |  | | | - | | | ++ | | |  | | | ++ | |  |  |  |  |  |
|  | **Q** | | | - | | |  | | | ++ | | | - | | | ++ | |  |  |  |  |  |
| **WDV-B** | **K** | | |  | | | + | | | - | | | + | | |  | |  |  |  |  |  |
|  | **N** | | | ++ | | |  | | | - | | | ++ | | | - | |  |  |  |  |  |
| Random II | | | 1 | 2 | | 3 | 4 | | 5 | 6 | | 7 | 8 | | 9 | 10 | | 11 | | 12 | 13 | 14 |
| **A** 92+91 | | | + | + | | + | + | | + | + | | + | + | | + | + | | + | | + | + | + |
| **D** 30+94 | | | - |  | |  |  | |  | - | | - | - | | + | - | | - | | - | - | - |
| **F** 93+96 | | | - |  | |  |  | |  | + | | + | - | | - | + | | + | | + | - | + |


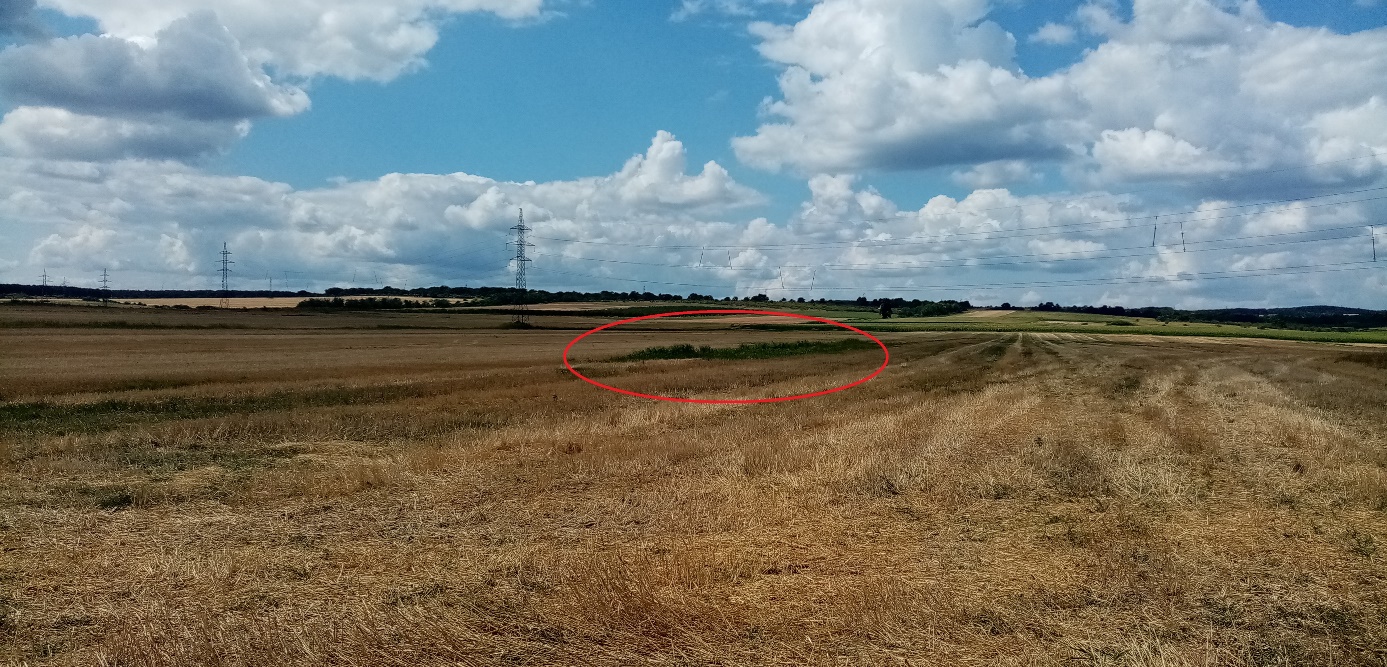


Figure S7. Site IX (Isztimér) on 25th July (event 24). The source of the only positive sample (*P. miliaceum*) from this site is marked.


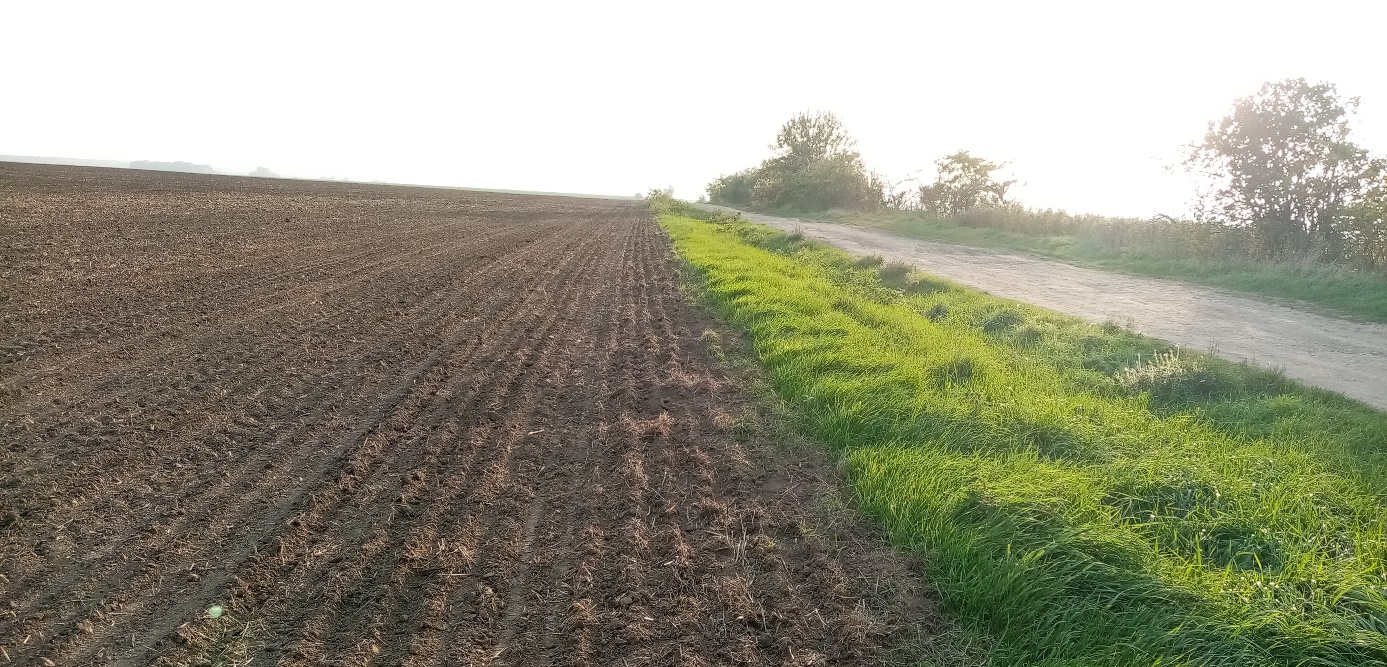


Figure S8. Site XI (Csákvár) on 26^th^ October (event 29).


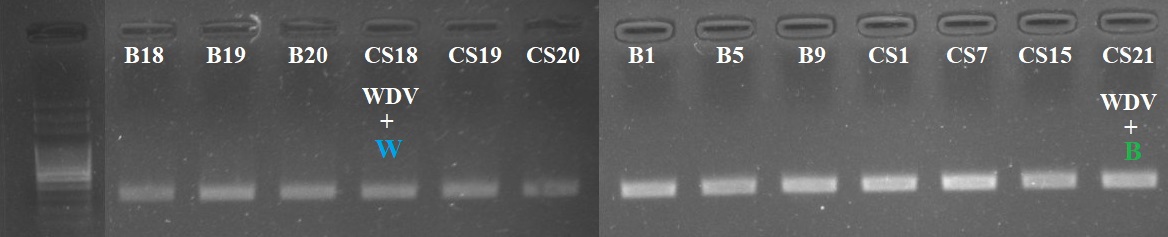


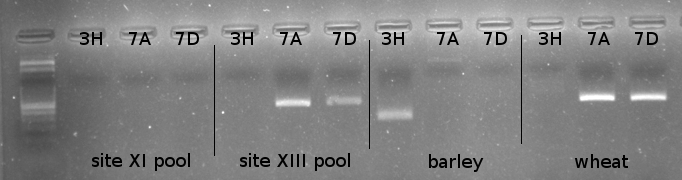


Figure S9. Confirmation of leafhopper and plant species identity with PCR at sites XI and XIII. Upper agarose gel photo shows the leafhoppers gathered at these two sites at event 31 and 33 (B=Bakonykúti, CS=Csákvár) tested with *P. alienus* specific primer pair 268F-435R. Sample CS18 and CS21 were positive for WDV (wheat and barley strain, respectively), other samples were negative and randomly selected. Lower gel shows the PCR test of plant pools collected from the tables at event 28 and 32. MBK primer pair 3H is barley genome, primer pairs 7A and 7D are wheat genome specific. At site XIII, wheat identity is confirmed. At site XI, plant sample was neither wheat nor barley and was further barcoded for ITS and ETS genes, and results showed that it is self-sown *Triticum* sp.


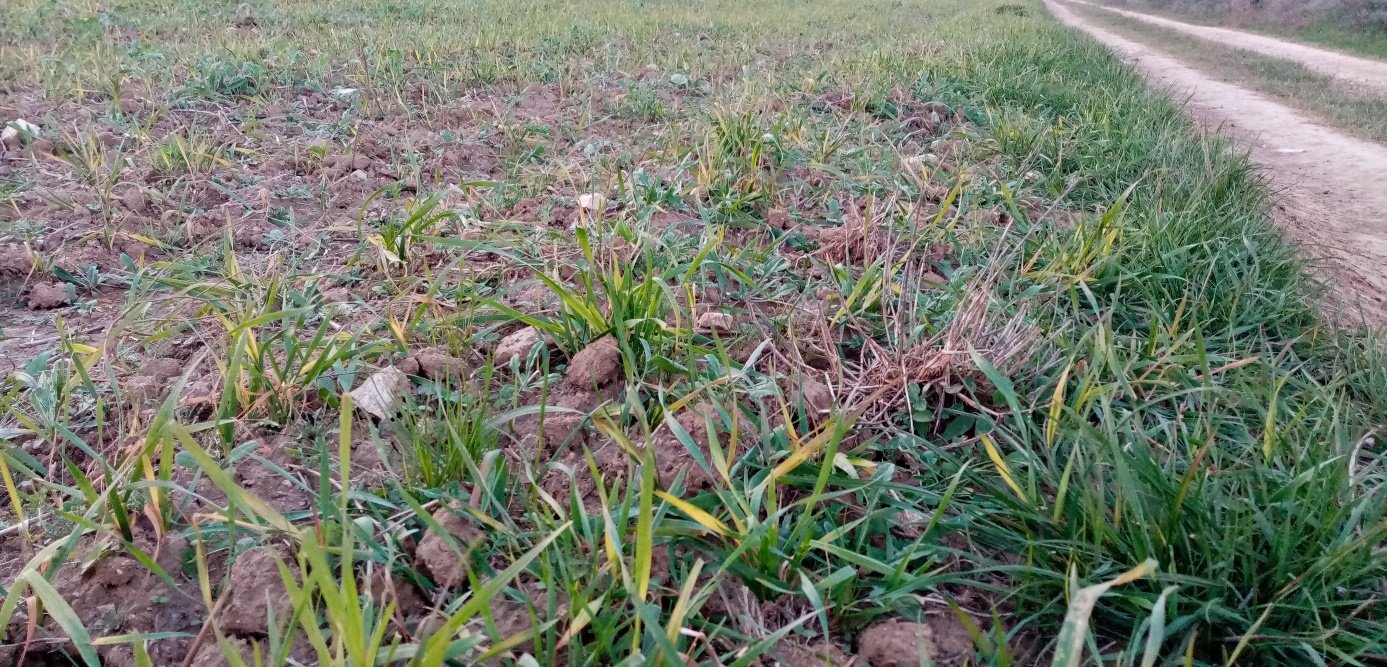


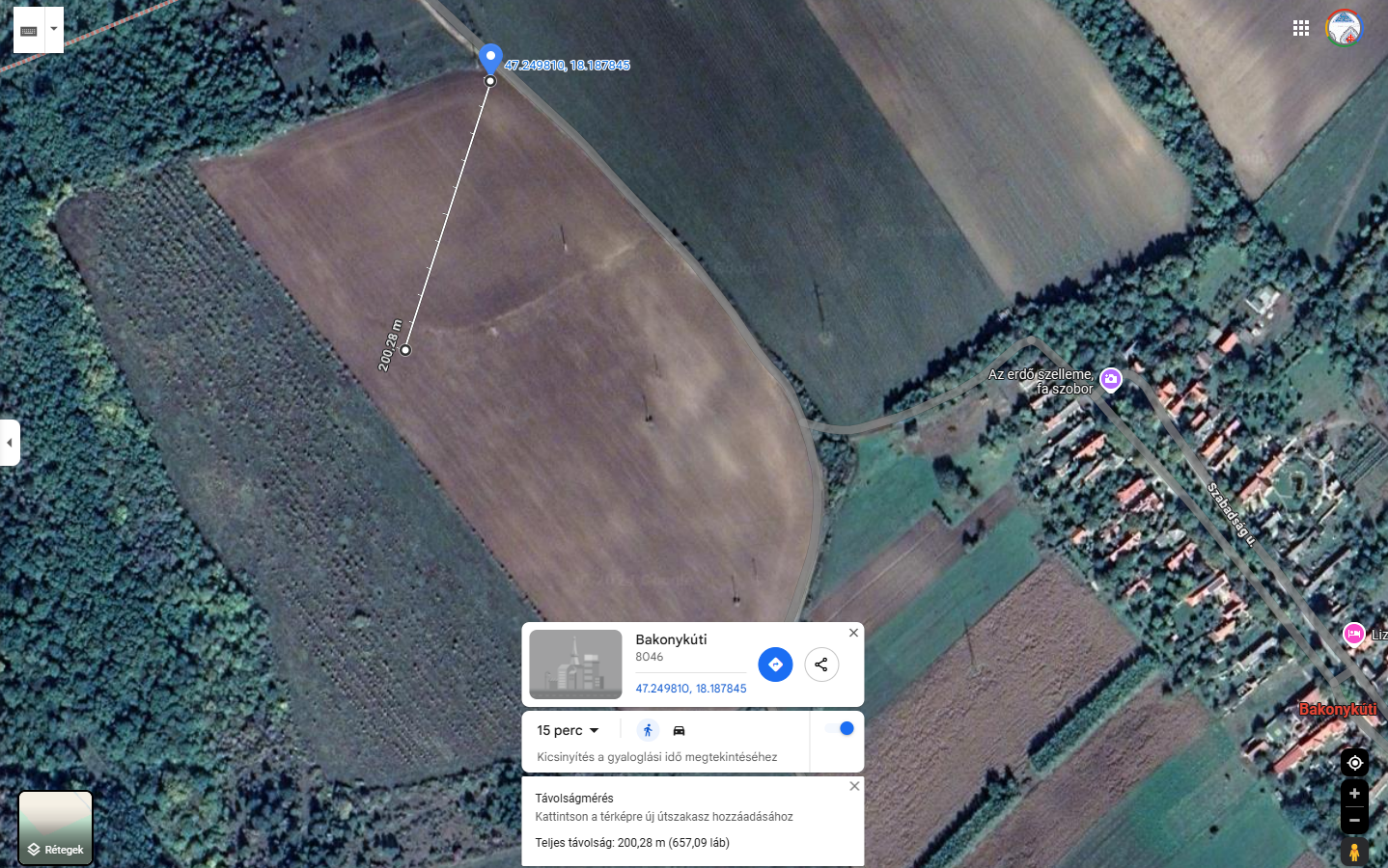


Figure S10. Site XIII (Bakonykúti) on 26^th^ October (event 33). Winter wheat field near the village showed heavy WDV symptoms (leaf yellowing, growth retention) through the whole field. 20 plant samples were gathered on a diagonal line across the field, 10 steps in between the sampling points.

Table S6. Linear gradient sampling at site XIII (Bakonykúti) on 26^th^ October (event 33). Samples from 1 to 20 are listed. Dark orange (+++) represents strong bands on the agarose gel (Fig. 6), lighter orange (++; +) means weaker bands, white (-) represents negative result. The WDV-positive samples were tested with barley and wheat strain-specific primers, listed in the first column. Empty cells mean no test was performed.

|  | 1 | 2 | 3 | 4 | 5 | 6 | 7 | 8 | 9 | 10 | 11 | 12 | 13 | 14 | 15 | 16 | 17 | 18 | 19 | 20 |
| --- | --- | --- | --- | --- | --- | --- | --- | --- | --- | --- | --- | --- | --- | --- | --- | --- | --- | --- | --- | --- |
| **A** 92+91 | **-** | **-** | **+** | **+++** | **+++** | **++** | **-** | **+** | **++** | **-** | **-** | **-** | **-** | **-** | **++** | **+** | **+** | **-** | **-** | **+** |
| **D** 30+94 |  |  | **-** | **-** | **-** | **-** |  | **-** | **-** |  |  |  |  |  | **-** | **-** | **-** |  |  | **-** |
| **F** 93+96 |  |  | **-** | **-** | **-** | **-** |  | **-** | **-** |  |  |  |  |  | **-** | **-** | **-** |  |  | **-** |
| **H** 87+91 | **-** | **-** | **-** | **-** | **+** | **+** |  | **-** | **+** |  |  |  |  |  | **-** |  |  |  |  |  |
| **O** 30+10 |  |  |  |  | **+** | **-** |  | **-** | **+** |  |  |  |  |  |  |  |  |  |  |  |
| **N** 93+10 |  |  |  | **-** | **+** | **-** |  | **-** | **-** |  |  |  |  |  | **-** | **-** |  |  |  | **-** |
| **J1** 30+31 |  |  |  | **-** | **-** | **-** |  | **-** | **-** |  |  |  |  |  | **-** | **-** |  |  |  | **-** |
| **Y** 122+123 |  |  |  |  | **-** | **-** |  | **-** | **-** |  |  |  |  |  |  |  |  |  |  |  |
| **X** 103+104 |  |  |  |  | **-** | **-** |  | **-** | **-** |  |  |  |  |  |  |  |  |  |  |  |
| **I** 88+91 | **-** | **-** | **-** | **-** | **-** | **-** |  | **-** | **-** |  |  |  |  |  | **-** | **-** |  |  |  | **-** |
| **Q** 9+91 |  |  |  | **-** | **-** | **-** |  | **-** | **-** |  |  |  |  |  | **-** | **-** |  |  |  | **-** |
| **L2** 30+90 |  |  |  | **-** | **-** | **-** |  | **-** | **-** |  |  |  |  |  | **-** | **-** |  |  |  | **-** |

Table S7. Result of the comparison of the newly sequenced WDV genomes and the references, acquired from NCBI BLASTn. Coverage (cov) and identity (id) of the sequences are presented in %.

|  | H07 | | vteny | | K1 | | K14 | | Pula | |
| --- | --- | --- | --- | --- | --- | --- | --- | --- | --- | --- |
|  | cov (%) | id (%) | cov (%) | id (%) | cov (%) | id (%) | cov (%) | id (%) | cov (%) | id (%) |
| H07 | 100 | 100 | 100 | 99.01 | 100 | 99.09 | 95 | 85.13 | 94 | 85.19 |
| vteny | 97 | 99.01 | 100 | 100 | 98 | 99.39 | 93 | 85.1 | 91 | 85.17 |
| K1 | 100 | 99.09 | 100 | 99.39 | 100 | 100 | 95 | 85.36 | 94 | 85.42 |
| K14 | 93 | 85.13 | 94 | 85.1 | 94 | 85.37 | 100 | 100 | 98 | 99.48 |
| Pula | 94 | 85.19 | 94 | 85.17 | 94 | 85.42 | 100 | 99.48 | 100 | 100 |


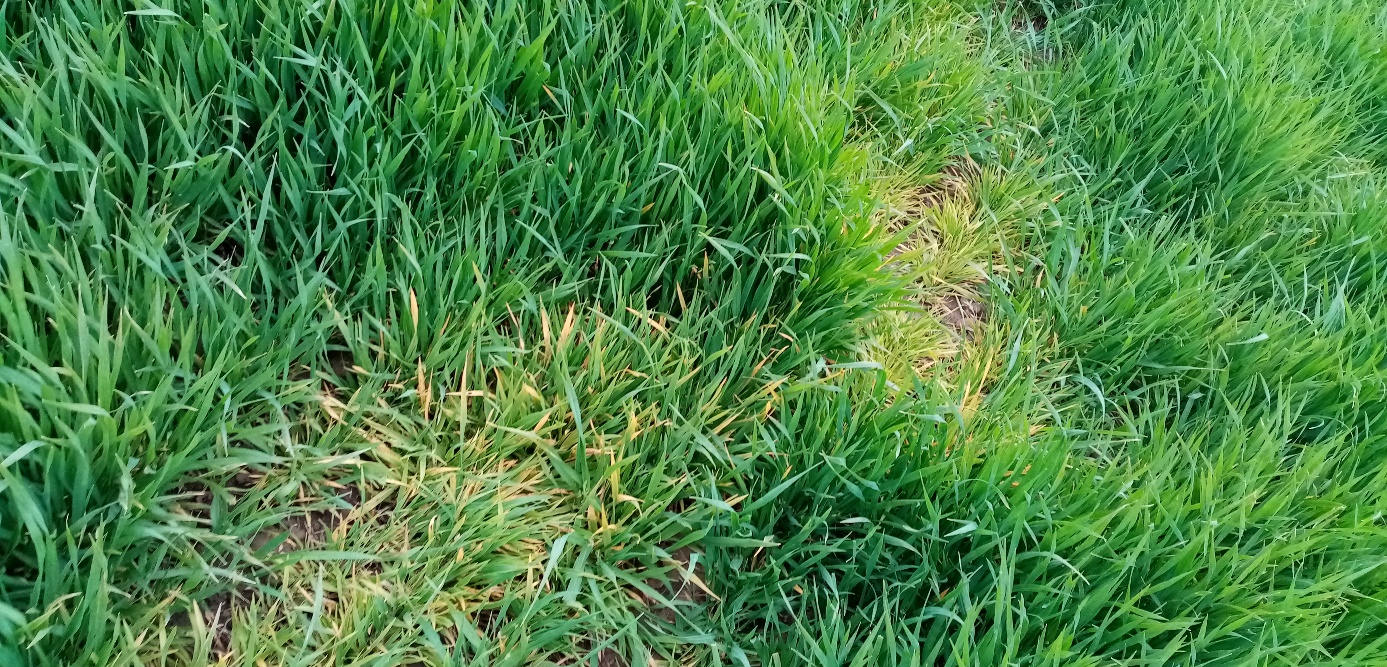

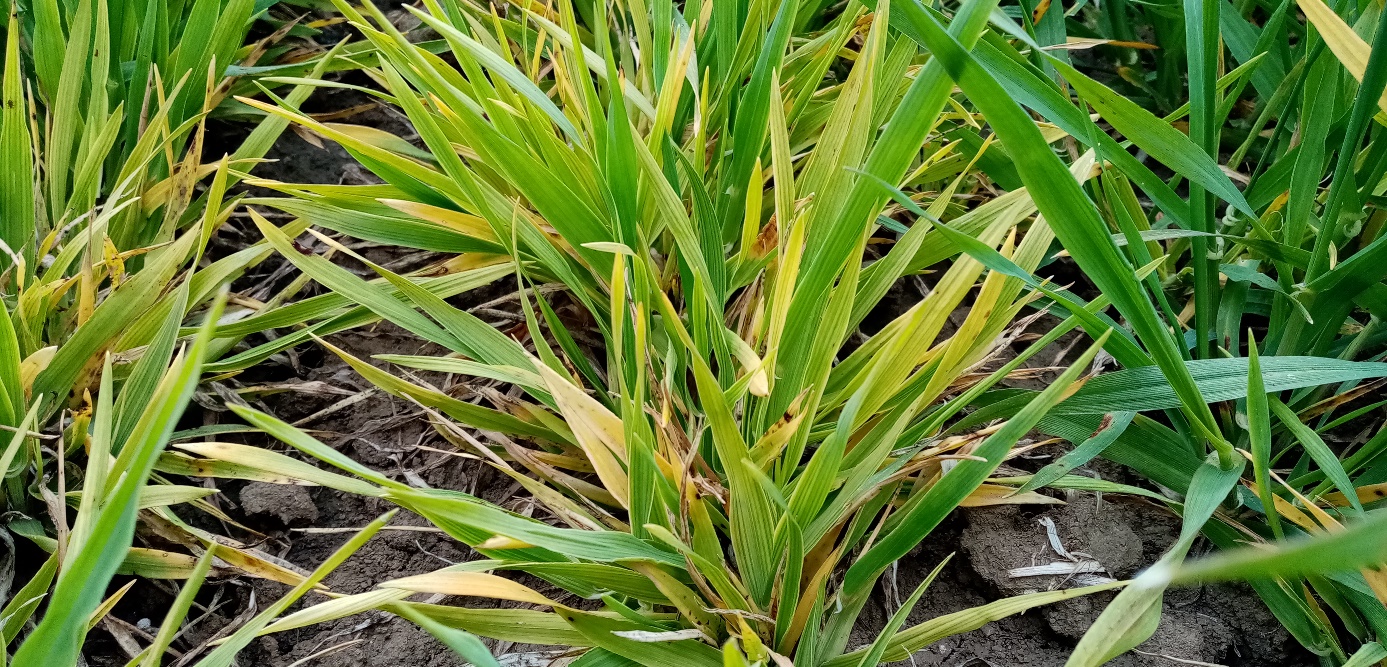

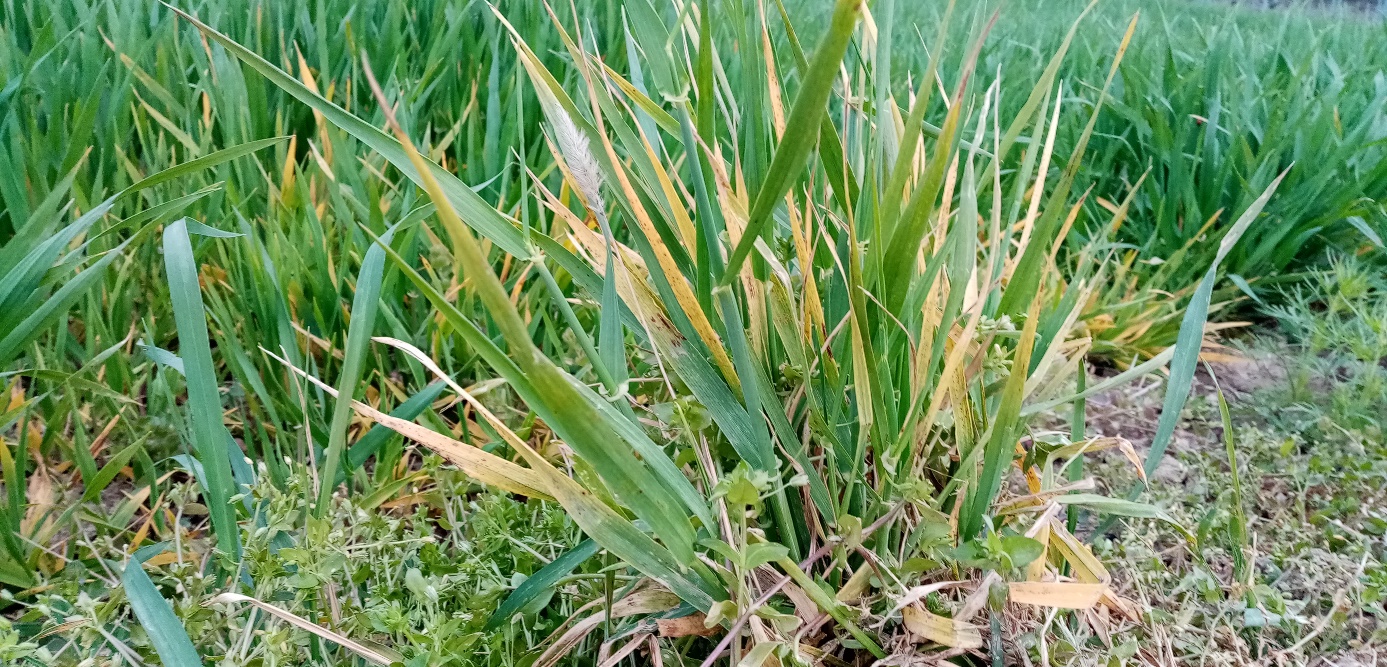
Figure S11. Apparently characteristically symptomatic wheat (top) and barley (bottom) from site IIIc (Vörösmart) and site IIIb (Csúza) on 10^th^ April, 2023 (sampling event 3 and 2, respectively). Streaking, dwarfing and yellowing are visible, although PCR tests were negative for WDV.


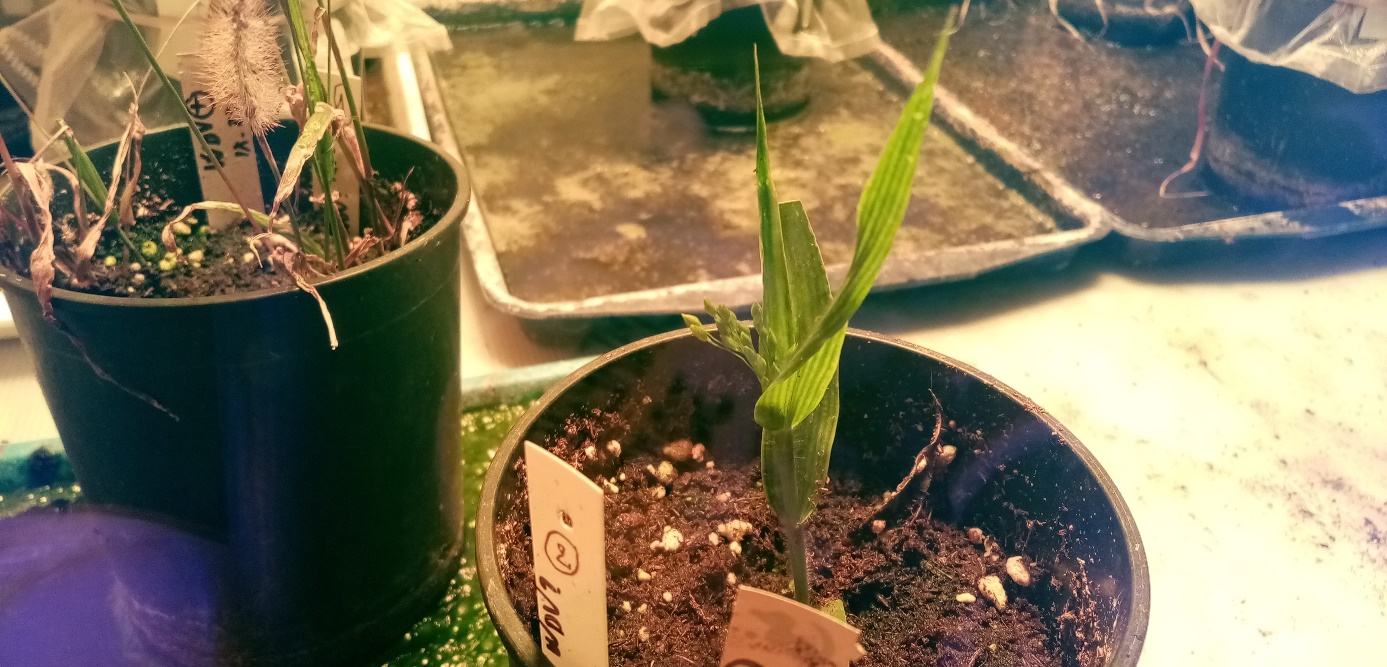


Figure S12. A potted specimen of *P. miliaceum* from sampling event 22 after 1 week of cultivation in the climate chamber of our institute.


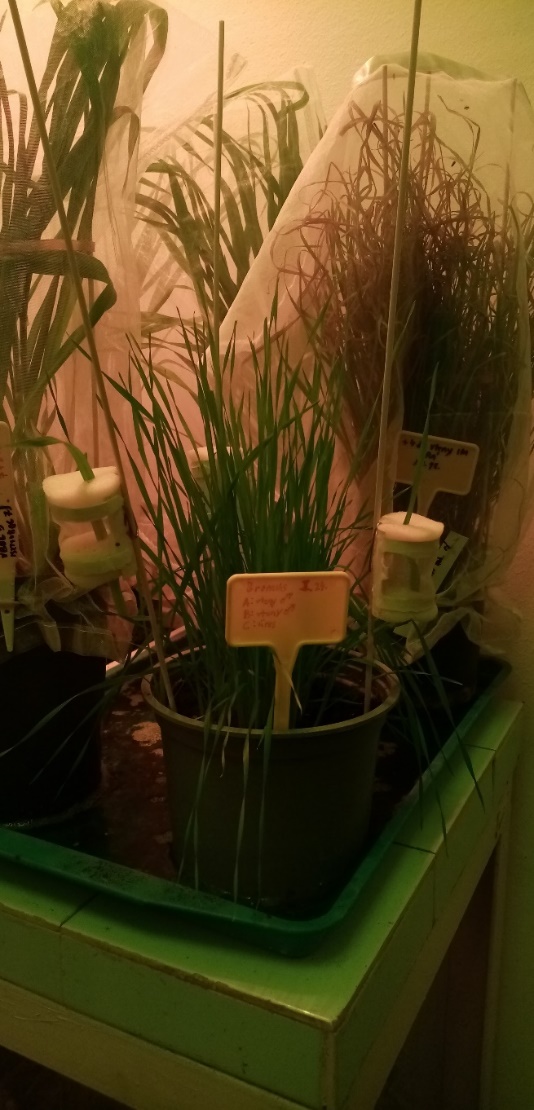


Figure S13. Microisolator-cage infection of downy brome by virus-carrying leafhoppers. In the back, soft brome is hosting a rearing for leafhoppers.


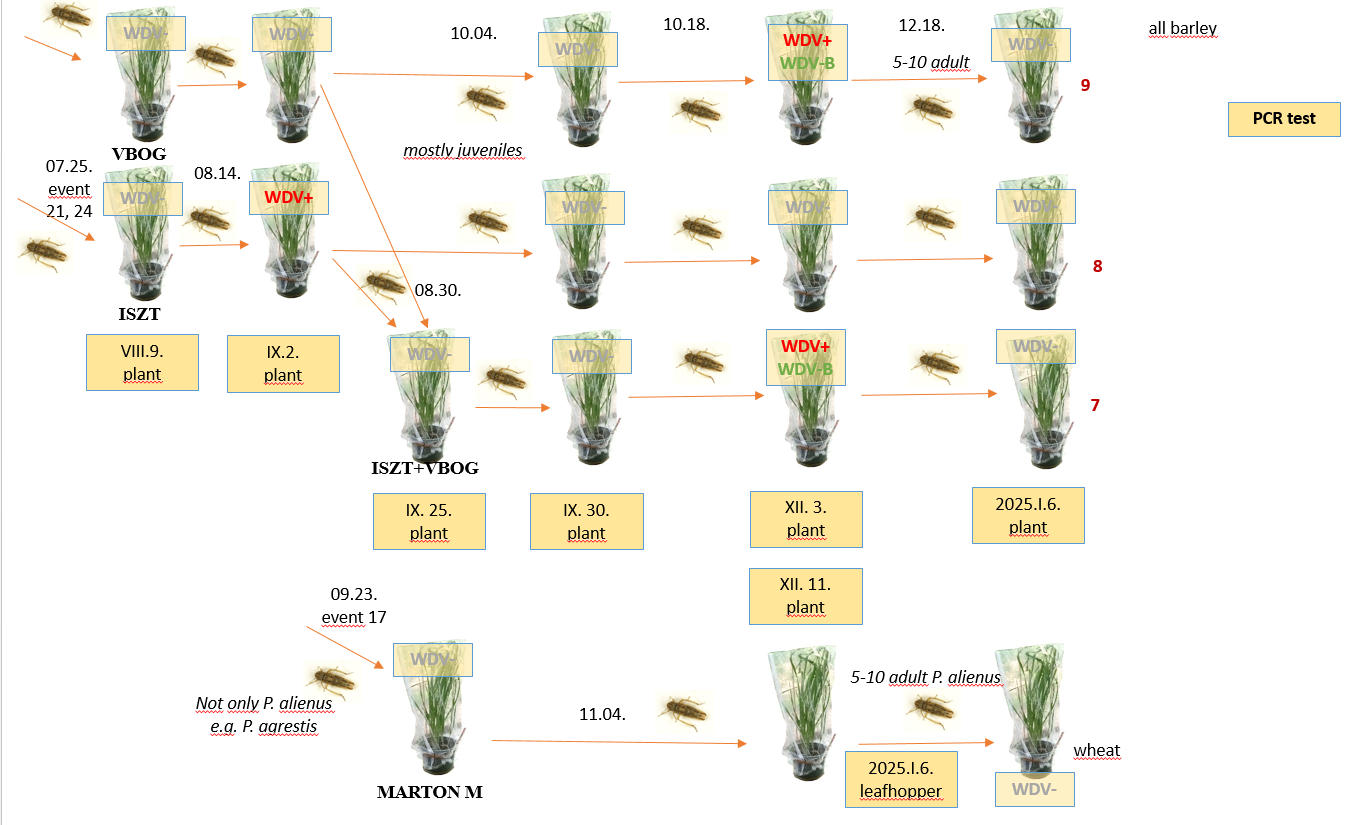


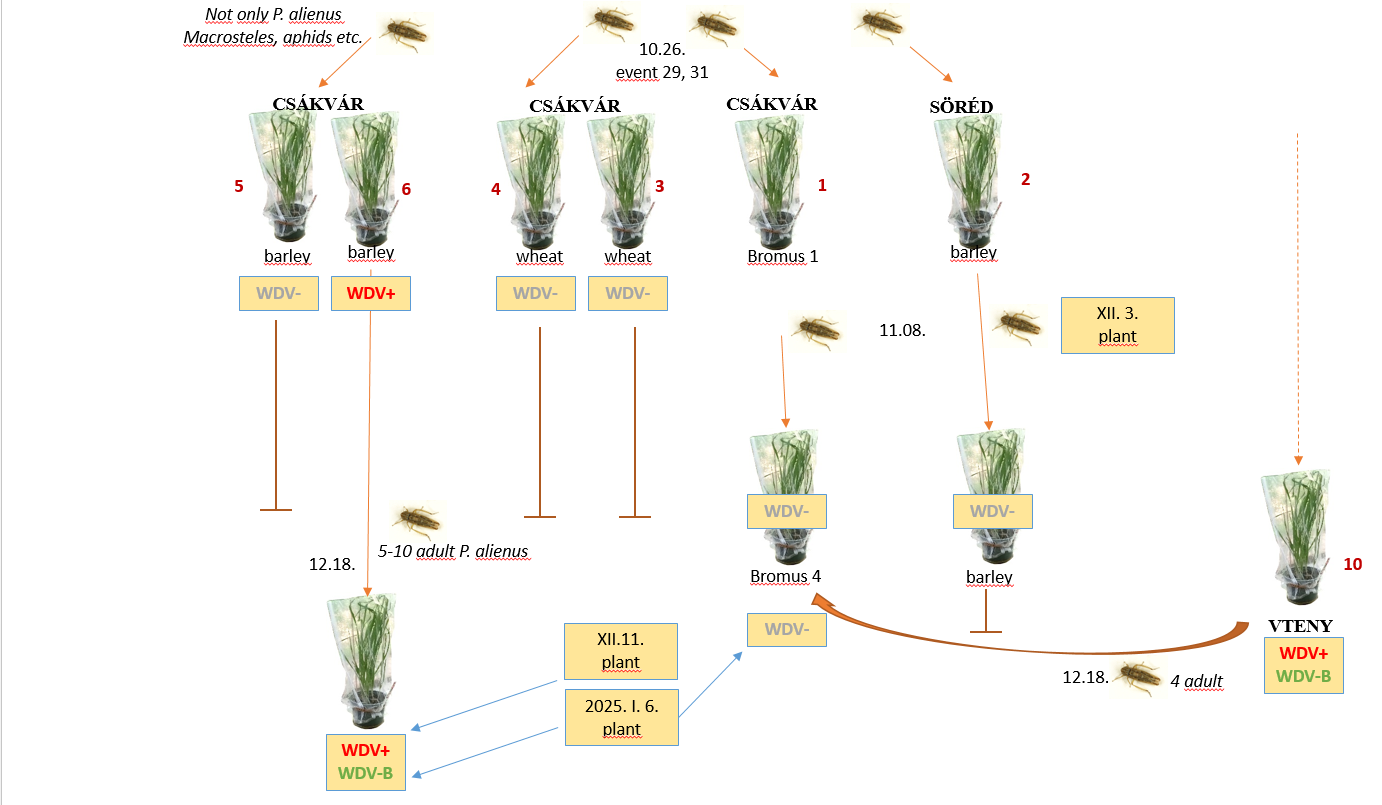


Figure S14. Time-schedule of the in-house leafhopper rearings. PCR testing of WDV are indicated with yellow boxes.

Ali, M., Polgari, D., Sepsi, A., Kontra, L., Dalmadi, A., Havelda, Z., Sagi, L. & Kis, A. (2024) Rapid and cost-effective molecular karyotyping in wheat, barley, and their cross-progeny by chromosome-specific multiplex PCR. Plant Methods 20: 37. doi: 10.1186/s13007-024-01162-x

Alonso, A., Bull, R. D., Acedo, C. & Gillespie, L. J. (2014) Design of plant-specific PCR primers for the ETS region with enhanced specificity for tribeBromeaeand their application to other grasses (Poaceae). Botany 92: 693-699. doi: 10.1139/cjb-2014-0062

Fulop, D., Szita, E., Gerstenbrand, R., Tholt, G. & Samu, F. (2019) Consuming alternative prey does not influence the DNA detectability half-life of pest prey in spider gut contents. PeerJ 7: e7680. doi: 10.7717/peerj.7680

Kuzmina, M. L., Braukmann, T. W. A., Fazekas, A. J., Graham, S. W., Dewaard, S. L., Rodrigues, A., Bennett, B. A., Dickinson, T. A., Saarela, J. M., Catling, P. M., Newmaster, S. G., Percy, D. M., Fenneman, E., Lauron-Moreau, A., Ford, B., Gillespie, L., Subramanyam, R., Whitton, J., Jennings, L., Metsger, D., Warne, C. P., Brown, A., Sears, E., Dewaard, J. R., Zakharov, E. V. & Hebert, P. D. N. (2017) Using herbarium-derived DNAs to assemble a large-scale DNA barcode library for the vascular plants of Canada. Appl Plant Sci 5: doi: 10.3732/apps.1700079

Omelchenko, D. O., Krinitsina, A. A., Kasianov, A. S., Speranskaya, A. S., Chesnokova, O. V., Polevova, S. V. & Severova, E. E. (2022) Assessment of ITS1, ITS2, 5′-ETS, and trnL-F DNA Barcodes for Metabarcoding of Poaceae Pollen. Diversity 14: doi: 10.3390/d14030191

Starr, J. R., Harris, S. A. & Simpson, D. A. (2003) POTENTIAL OF THE 5' AND 3' ENDS OF THE INTERGENIC SPACER (IGS) OF rDNA IN THE CYPERACEAE: NEW SEQUENCES FOR LOWER-LEVEL PHYLOGENIES IN SEDGES WITH AN EXAMPLE FROM UNCINIA PERS. Int. J. Plant Sci. 164: 213–227.
